# Identifying environmental factors affecting the microbial community composition on outdoor structural timber

**DOI:** 10.1007/s00253-024-13089-3

**Published:** 2024-03-06

**Authors:** Lauritz Schrader, Jochen Trautner, Christoph C. Tebbe

**Affiliations:** 1 Thünen Institute of Wood Research, Leuschnerstraße 91, 21031 Hamburg, Germany; 2 Thünen Institute of Biodiversity, Bundesallee 65, 38116 Brunswick, Germany

**Keywords:** Timber wood, Biodegradation, Fungal community composition, Bacterial community composition, *Perenniporia meridionalis*

## Abstract

**Abstract:**

Timber wood is a building material with many positive properties. However, its susceptibility to microbial degradation is a major challenge for outdoor usage. Although many wood-degrading fungal species are known, knowledge on their prevalence and diversity causing damage to exterior structural timber is still limited. Here, we sampled 46 decaying pieces of wood from outdoor constructions in the area of Hamburg, Germany; extracted their DNA; and investigated their microbial community composition by PCR amplicon sequencing of the fungal ITS2 region and partial bacterial 16S rRNA genes. In order to establish a link between the microbial community structure and environmental factors, we analysed the influence of wood species, its C and N contents, the effect of wood-soil contact, and the importance of its immediate environment (city, forest, meadow, park, respectively). We found that fungal and bacterial community composition colonising exterior timber was similar to fungi commonly found in forest deadwood. Of all basidiomycetous sequences retrieved, some, indicative for *Perenniporia meridionalis*, *Dacrymyces capitatus*, and *Dacrymyces stillatus*, were more frequently associated with severe wood damage. Whilst the most important environmental factor shaping fungal and bacterial community composition was the wood species, the immediate environment was important for fungal species whilst, for the occurrence of bacterial taxa, soil contact had a high impact. No influence was tangible for variation of the C or N content. In conclusion, our study demonstrates that wood colonising fungal and bacterial communities are equally responsive in their composition to wood species, but respond differently to environmental factors.

**Key points:**

• *Perenniporia meridionalis and Dacrymyces are frequently associated with wood damage*

• *Fungal community composition on timber is affected by its surrounding environment*

• *Bacterial community composition on structural timber is affected by soil contact*

**Supplementary Information:**

The online version contains supplementary material available at 10.1007/s00253-024-13089-3.

## Introduction

Timber is a building material with many positive properties, i.e. its renewability, workability, high strength-to-weight ratio, and low environmental impact compared to other materials like steel or concrete (Gustavsson et al. 2006; van Niekerk et al. 2022; Wimmers 2017). Because of its potential to mitigate climate change, use of timber is promoted in national and international policies (Bundesregierung 2021; European Commission 2018), which will most likely lead to increased use. However, the susceptibility of timber wood to biodegradation is a major challenge for its outdoor usage. Especially, physically stressed wooden constructions, i.e. bridges or playground equipment, which are prone to degradation due to permanent exposition to precipitation, could become hazardous (Arpaci et al. 2021; Oberhofnerová et al. 2017).

In general, wood degradation can be caused by various organisms including insects, and a high diversity of different fungi and bacteria (Blanchette et al. 1990). Numerous fungal and bacterial species can utilise the different polysaccharides and lignins of wood as a carbon and energy sources (Huckfeldt and Rehbein 2012). For fungal decomposition, depending on the emerging macroscopic properties, white rot, brown rot, or soft rot can be distinguished (Schwarze 2007). Based on the micromorphological decay patterns of the cell wall, two different types of wood-degrading bacteria can also be identified, namely, tunnel and erosion bacteria (Singh et al. 2016). The actual diversity and environmental variation of fungal and bacterial communities involved in the decomposition processes is still poorly understood, and therefore, it is yet not possible to predict microbial colonisation of timber and suggest, e.g. based on microbial indicators, potential protection strategies. In fact, as we can learn from forest ecology, dead wood is usually inhabited by complex fungal and bacterial communities rather than by single taxa (Baldrian et al. 2016; Leonhardt et al. 2019; Moll et al. 2018).

Some wood decaying microorganisms may already be present in the living tree as latent propagules (Parfitt et al. 2010), but, in contrast to decomposing deadwood in forest ecosystems, timber wood is usually subjected to a heat drying processing. Even though solid scientific evidence is still missing, it was assumed that such heat treatments would be lethal to most if not all microorganisms present in the wood (Embacher et al. 2023), and thus, timber wood decaying microorganisms may predominantly originate from the surrounding environment, i.e. air, soil, water bodies, or insects (Birkemoe et al. 2018; Johnston et al. 2016). Considering the high microbial diversity which exists in soil, contact of outdoor timber wood could create many opportunities for microbial colonisation in the transitional zone from below to above ground. In fact, it has been shown that these regions are more prone to biological degradation, probably due to the environmental conditions with sufficient moisture and oxygen supply with favourable growth conditions for aerobic microbial decomposers (Goodell et al. 2020; Huckfeldt and Rehbein 2012). In contrast, for timber with no contact to soil, the lack of moisture and the greater potential for desiccation after rainfall events would limit microbial colonisation of such material (Goodell et al. 2020). The taxa and diversity of wood degrading organisms which may prevail under such different conditions could be very different and also dependent on the tree from which the timber was obtained.

The aim of this study was to characterise the composition of fungal and bacterial communities colonising decomposing outdoor structural timber and establish links between their occurrence and some relevant environmental variables. For this purpose, we collected a total of 46 samples of outdoor timber wood constructions (bridges, playground equipment) which showed white, brown, or soft rot symptoms. From each sample a subsample from the surface and one from the interior was taken. Then, we extracted DNA from the respective wood material separately, and PCR amplified with phylogenetically highly conserved primers the fungal ITS2 region and partial bacterial 16S rRNA genes for a subsequent DNA-sequencing and bioinformatic analysis indicating their taxonomic affiliation. For each piece of wood, we determined the tree species from which the wood was obtained, measured its carbon and nitrogen contents, analysed if the wood had been in contact with soil or not, and assigned its immediate environment to one of four categories, i.e. city, forest, meadow, and park, respectively. Furthermore, it was tested whether the predominant type of fungal decay (white, brown, or soft rot) had an effect on the associated bacterial community composition.

## Materials and methods

### Field sampling

Samples were taken from the city region of Hamburg (Germany) and its surrounding. In total, 46 wooden parts were collected from various outdoor constructions, including bridges, huts, park benches, and playground equipment. All samples showed signs of decay. For the sampling a two-step procedure was implemented: in the first step, several square centimetres of the wooden surface were removed with a scalpel. In the second step, two to four holes, depending on the extent of decay, were drilled with a cordless drill (drill bit: 6 × 60 mm). Both carvings and drill dust per piece of wood were collected in two separate plastic bags, kept cold during transportation, and frozen at − 20 °C until further processing. In between samples, all tools were first rinsed with ethanol (70%) and then flamed for sterilisation.

All samples were taken from constructions managed and maintained by various institutions of the city of Hamburg, for which permission for sampling was obtained (Supplemental Material, Table S1). For 28 samples no reliable information on the used wood species was available. In these cases, an extra sample (~ 1 × 1 × 1 cm) was taken from the non-decayed part of the affected wood piece using a Japanese saw and chisel. For identifying wood species at least to genus level, thin transverse and radial sections were cut with a razor blade and examined under a transmitted light microscope (100–400 × magnification). Based on the microscopic anatomical features wood species of all samples could be assigned to genus or species level.

### Wood chemical properties

For measurements of carbon C and nitrogen N content, the samples were ground with a semi-automatic vibrating mill (Herzog, Osnabrück, Germany). Each sample was milled for 45 s in a tungsten carbide milling bowl. Between samples all tools used were first rinsed with distilled water and then blow dried with compressed air. The generated powders were then transferred to sterile glass tubes and oven dried at 103 °C for 24 h. For the analysis of one sample 10 mg (± 1 mg) of wood powder together with 45 mg (± 5 mg) of tungsten trioxide (WO_3_, Elementar, Langenselbold, Germany) were transferred into a tin capsule which was then sealed manually. The capsules were then loaded into a vario EL CUBE (Elementar, Langenselbold, Germany) which was set on modus CHNS. Briefly, the samples were burnt at approximately 900 °C and resulting gaseous reaction products (N_2_, CO_2_, H_2_O, and SO_2_) separated from each other by three adsorption columns connected in series. The substances were successively desorbed again by heat and measured with a thermal conductivity detector. The addition of tungsten trioxide prevents the formation of non-volatile sulphates and binds interfering alkali and alkaline earth elements. Measurements were carried out in duplicates. Only samples taken from the inside were chosen for measuring C and N contents. For one sample (76), no measurements could be executed due to insufficient wood material.

### DNA extraction, sequencing, and qPCR

DNA was extracted from 50 mg dry weight of wood using the DNeasy PowerSoil Pro Kit (Qiagen, Hilden, Germany) according to the manufacturers’ instructions. Cell disruption was accomplished with three bead beating runs of 30 s at 6.5 m s^−1^ using a FastPrep-24 system (MP Biomedicals, Eschwege, Germany) with the tubes containing eight 2-mm stainless steel spheres. The quantity of genomic DNA was measured with a NanoDrop2000 spectrophotometer (Thermo Fisher Scientific, Darmstadt, Germany). Extracts were kept at − 20 °C until they were sent to a sequencing facility (StarSEQ, Mainz, Germany) where amplification of target regions, library preparation, and paired-end sequencing (2 × 300 bp) on an Illumina MiSeq platform was executed. For determination of bacterial diversity, the V4 region of the 16S rRNA gene was amplified using the primer pair 515F (5′-GTG**Y**CAGCMGCCGCGGTAA-3′) and 806R (5′-GGACTACNVGGGTWTCTAAT-3′) (Parada et al. 2016), whilst for fungal diversity the ITS2 region was targeted using primers ITS3 (5′-GCATCGATGAAGAACGCAGC-3′) and ITS4 (5′-TCCTCCGCTTATTGATATGC-3′), respectively (White et al. 1990). The protocols of the service company are based on publications of Caporaso et al. (2012), Apprill et al. (2015), and White et al. (1990). The raw DNA sequence data have been deposited at the European Nucleotide Archive (ENA) (Project Accession Number: PRJEB63071).

Quantitative real-time PCR (qPCR) was used to estimate, based on gene copy numbers per g of dry weight timber, the abundances of fungi and bacteria using a Bio-Rad CFX96 Touch Real time PCR Detection System (Biorad, Feldkirchen, Germany). For fungi, amplification of the ITS2 region was done using the primer pair gITS7 (5′-GTGARTCATCGARTCTTTG-3′) and ITS4 (Ihrmark et al. 2012; White et al. 1990) and for bacteria the amplification of the V4 region was done with the primer pair 515F and 806R (Parada et al. 2016). All reactions had a total volume of 20 μl composed of 10 μl Maxima SYBR Green qPCR Master Mix (Thermo Fisher Scientific, Darmstadt, Germany), 0.1 μl of each primer (100 μM), 1–5 μl of template containing approximately 10 ng DNA, and the remaining volume was filled with DNA/RNase-free water. Cycling conditions for fungal DNA were as follows: initial degeneration for 2 min at 95 °C and 30 cycles of 95 °C for 30 s, 56 °C for 30 s, and 72 °C for 30 s. For bacterial DNA the cycling conditions consisted of 10 min at 95 °C followed by 39 cycles of 95 °C for 15 s and 52 °C for 1 min. Standard curves were prepared from tenfold dilutions of DNA obtained from pure cultures of *Fusarium culmorum* and *Bacillus subtilis*. PCR efficiencies range from 90.9 to 95.6% for fungi and from 93.3 to 94.1% for bacteria with *R*^2^ ≥ 0.998 in all cases.

### Bioinformatic analyses

All raw reads were reoriented and demultiplexed by the sequencing facility. Further processing was done with the QIIME2 bioinformatics pipeline (Bolyen et al. 2019). The bacterial forward and reverse reads were separately truncated based on quality scores, forward reads at position 230 and reverse reads due to lower quality at position 130. These were then filtered, denoised, and merged using the DADA2-plugin (maxEE: 2; Callahan et al. 2016). Amplicon sequence variants (ASV) were adopted and assigned with the SILVA 138 reference database (Quast et al. 2013; Yilmaz et al. 2014). The fungal reads were treated in a similar way with truncation of the forward reads at position 240 and reverse reads at 180 and then processed with DADA2. For annotation of fungal ASV the UNITE reference database (v. 9.0; Abarenkov et al. 2023; Kõljalg et al. 2020) was used, respectively. Dominant ASV were defined as those that had at least 10% relative abundance in one sample. The count tables and associated taxonomy information were exported for bacteria and fungi and imported to R. Eukaryote-associated ASV (mitochondria and chloroplasts) were removed from the 16S rRNA gene amplicon sequence dataset. Singletons and doubletons were removed from both datasets. Ecological traits (primary lifestyle and decay type) were assigned for fungal ASV based on genus-level identification using the FungalTraits database (v. 1.2; Põlme et al. 2020).

### Statistical analysis

The complete data analysis was carried out in R (v.4.2.1). Differences in absolute abundances were checked with the Welch’s *t*-test and significance was considered as *Ρ*-values less than 0.05. The α-diversity metrics were calculated based on unfiltered data (e.g. including singletons and doubletons) using ‘estimate_richness’ function of the ‘phyloseq’ package (v. 1.40.0; McMurdie and Holmes 2013). Statistical differences in α-diversity were examined using Wilcoxon signed rank test and significance was considered as *Ρ*-values less than 0.05. Stacked bar charts were created to visualise relative abundances of ASV found in each sample deploying the ‘phyloseq’ and ‘ggplot2’ packages (v. 3.3.6; Wickham et al. 2016). Due to the compositional nature of sequencing data following steps of the analyses were based on Gloor et al. (2017) and the online tutorial “Introduction to the Statistical Analysis of Microbiome Data in R” (Ollberding 2019). First, normalisation of read counts was performed by converting them to ratios through a centred log ratio (CLR) transformation. For a successful transformation it was necessary to assign a pseudocount of 1 to all zeros beforehand, because the logarithm of zero is undefined. Both was done using the ‘microbiome’ package (v. 1.18.; Lahti et al. 2017). Subsequently, principal component analyses (PCA) were conducted and plots were created based on Aitchison distance with the two principal components explaining most of the variation on the *x*- and *y*-axes, respectively (Aitchison 1983).

Permutational multivariate analyses of variance (PERMANOVA) were carried out to test whether environmental factors (e.g. wood species, C and N content, soil contact, surrounding area, decay type) have an effect on the fungal and bacterial community compositions (Anderson 2001). For this purpose, the ‘adonis2’ function from the ‘vegan’ package (v. 2.6.2; Oksanen et al. 2022) with 999 permutations was used. Statistical significance was considered as *Ρ*-values with less than 0.05. Differential abundance testing was done using the ‘Aldex2’ package (v. 1.28.1; Fernandes et al. 2013, 2014) to identify ASV that are associated with certain environmental factors. First, a CLR transformation is applied to the count data and subsequently a distribution of CLR values generated by 128 Monte Carlo samplings. Then, effects were tested with a Welch’s *t*, Wilcoxon rank-sum, or Kruskal–Wallis test depending on the environmental factor using the ‘aldex’ function. Statistically significant differences were considered as Benajmini-Hochberg corrected *Ρ*-values less than 0.05 (Benjamini and Hochberg 1995).

The influence of chemical wood properties, i.e. carbon (C), nitrogen (N), and C/N ratio, on β-diversity was assessed by checking what portion of data variation each factor explains. For that, we performed variation partition analyses on clr-transformed ASV using the ‘varpart’ function from the vegan package (Oksanen et al. 2022). Statistical significance was tested with redundancy analyses with 999 permutations. Due to unbalanced sample sizes of wood species no reliable statistical tests could be executed to check for differences in C, N, and C/N ratio between wood species.

Network analysis was performed to indicate possible links between fungal and bacterial ASV using the ‘NetCoMi’ package (v.1.1.0; Peschel et al. 2021) For this, we first combined the count data from the interior samples of both domains and then filtered out all ASV with less than 1500 total counts to focus on abundant ASV, leaving 582 ASV for network construction. We then added a pseudocount to all zeros and performed a clr transformation to account for compositionality. Finally, we calculated pairwise Pearson correlation coefficient between all ASV. For plotting the network, a fast greedy algorithm and only correlation coefficients greater 0.75 were considered (Clauset et al. 2004).

### Cultivation, morphological identification, and Sanger sequencing

To evaluate questionable annotations of a dominant ASV, fruitbodies were collected from five damaged constructions. Spores and pores were measured using an AxioLab (Zeiss, Oberkochen, Germany). Cyanophilic reaction was tested using 3% lactic acid cotton blue and dextrinoid reaction was checked using Melzer’s reagent. All measurements were done in Melzer’s reagent and identification was based on Decock and Stalpers (2006). Pure cultures were then prepared from fruitbodies, grown on 4% malt extract agar and used for molecular identification. DNA was extracted from 50–100 mg of mycelium using the DNeasy Plant Mini Kit (Qiagen, Hilden, Germany). The quantity of genomic DNA was measured with a NanoDrop2000 spectrophotometer (Thermo Fisher Scientific, Darmstadt, Germany). Subsequently, the full ITS region was amplified using the ITS1/ITS4 primer pair (White et al. 1990). All reactions had a total volume of 50 μl composed of 25 μl 2X YourTaq PCR Master Mix (biotechrabbit GmbH, Berlin, Germany), 1 μl of each primer (10 μM), 1–5 μl of template containing approximately 10 ng DNA, and the remaining volume was filled with DNA/RNase-free water. Cycling conditions were as follows: initial degeneration for 5 min at 94 °C and 25 cycles of 94 °C for 30 s, 51 °C for 45 s, and 72 °C for 90 s followed by 15 min at 72 °C (Větrovský et al. 2016). Afterwards PCR products were checked by gel electrophoresis on a 1% agarose gel and then purified using the Monarch PCR & DNA Cleanup Kit (New England Biolabs GmbH, Frankfurt a. M., Germany). PCR products were sent to a sequencing facility (StarSEQ, Mainz, Germany) for Sanger sequencing. The forward and reverse reads of each sample were aligned using BioEdit (v.7.2) (Hall, 1999).

## Results

### Characteristics of the wood material

The wood material collected in this study originated from eight different tree species with most of them from *Quercus* spp. and *Robinia pseudoacacia* (Supplemental Table S1). Brown rot and white rot symptoms were collected in approximately equal numbers, and in addition, one sample showed soft rot symptoms. Generally, the C content of wood species ranged from 45.8% (*Abies alba*, brown rot) to 51.9% (*Pinus sylvestris* and *Picea abies*, both brown rot) and the N content from 0.16% (*Abies alba*, brown rot) to 0.45% (*Quercus* spp., brown rot). The lowest measured C:N ratio was 126 for brown rotted oak wood, whilst brown rotted fir wood had the highest C:N ratio of 286 (Supplemental Table S2).

### Fungal community composition

The average copy number from samples taken from the surface of the respective sampling area was 1.2 × 10^11^ gene copies per g wood material (dry weight) which is significantly higher (*ρ* = 0.001) compared to the samples from the interior with 3.1 × 10^10^ (Supplemental Table S3).

For DNA sequencing, the 92 samples analysed in this study yielded 3.91 million reads of which 2.68 million passed the filtering process and led to the annotation of 3623 fungal ASV (Supplemental Table S4). A total of 87 singletons and doubletons were removed. The composition of fungal divisions of the remaining 3536 ASV comprised *Ascomycota* (64.1%), followed by *Basidiomycota* (18.7%), and other fungal divisions with less than 1% relative abundance, including *Mucoromycota*, *Rozellomycota*, *Chytridiomycota*, *Olpidiomycota*, and 13.8% remained unidentified.

**Fig. 1:**
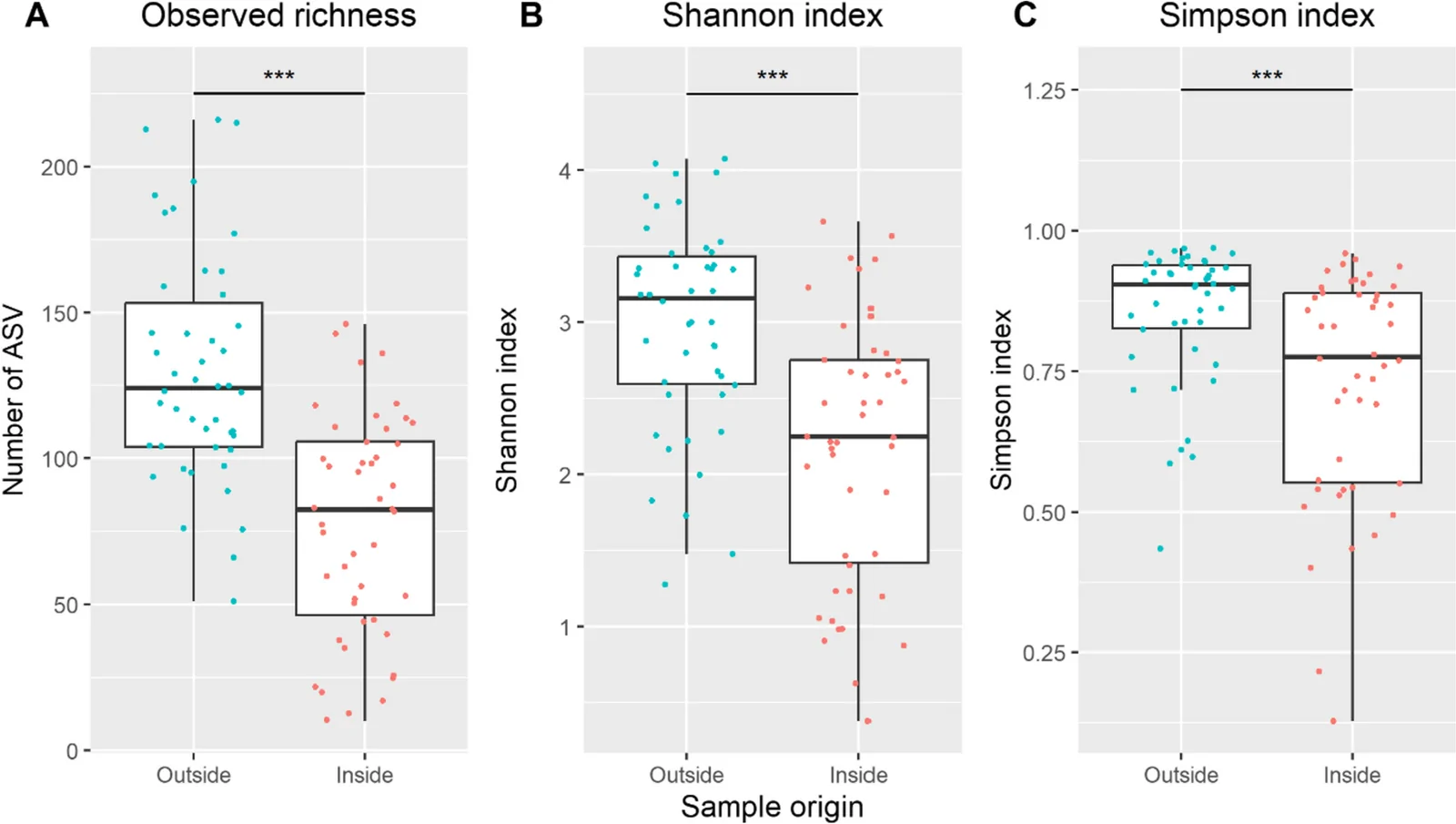
Diversity of fungal microbial communities based on ITS2 amplicon sequence variants (ASV) associated with timber wood damage, as sampled from the surface (outside) and inside material. Comparison of different diversity metrics. **A** Observed richness, **B** Shannon index, and **C** Simpson index of fungal ASV between samples taken from the outside and inside. The asterisks indicate the level of statistical significance (observed: Wilcoxon signed rank test, *ρ* < 0.001; Shannon: Wilcoxon signed rank test, *ρ* < 0.001; Simpson: Wilcoxon signed rank test, *ρ* < 0.001)

More fungal ASV were exclusively detected on the wood surfaces (1620 ASV, with 1110 *Ascomycota*, 207 *Basidiomycota*) as compared to the interior (750 ASV, with 419 *Ascomycota*, 175 *Basidiomycota*). The remaining 1166 ASV (737 *Ascomycota*, 279 *Basidiomycota*) were found in both surface and interior samples. A higher richness was detected for samples taken from the surface (Fig. 1). Likewise, different α-diversity metrics were seen for both the Shannon index (*ρ* < 0.001) and Simpson index (*ρ* < 0.001), being significantly higher for surface as compared to interior samples.

The annotation of the suspected ecological traits of detected fungal ASV suggested that most belonged to wood saprotrophs, followed by soil saprotrophs, animal parasites, and plant pathogens (Supplemental Fig. S1). Basidiomycetous wood saprotrophs causing white rot were mostly associated with hardwood species (e.g. oak, black locust), whilst brown rot fungi were more present on softwood species (e.g. pine, spruce, Douglas fir, silver fir, European larch). Ascomycetous wood saprotrophs causing soft rot or mould were found equally on hard and softwood species (Supplemental Fig. S2).

In total 47 dominant ASV could be assigned to *Basidiomycota* of which 31 were suspected wood saprotrophs. Comparing the interior with the exterior, there was not much difference in dominant ASV found, with one or two accounting for most of the relative abundance of an individual sample (Supplemental Fig. S3). Outstanding ASV affiliated with *Perenniporia vanhulleae* were found on six oak wood samples. Furthermore, ASV indicative for three *Dacrymyces* species colonising 14 samples were detected across seven different wood species. Amongst the *Ascomycota*, 82 dominant ASV could be assigned. Although the number of dominant ASV was higher compared to *Basidiomycota*, they account for a much smaller proportion of relative abundance per sample, whilst the “Others” category accounts for a much higher proportion (Supplemental Fig. S4).

The questionable annotation of the dominant fungal ASV *P. vanhulleae* was re-analysed based on morphological and molecular technics (see the “Cultivation, morphological identification, and Sanger sequencing” section), which revealed that the actual species found was in fact *Perenniporia meridionalis* (Supplemental Table S5). The identity of *Dacrymyces stenosporus*, a species from New Zealand, could not be analysed in the same way, since the affected wood piece had been replaced by local managers before fruitbodies could be collected.

**Fig. 2:**
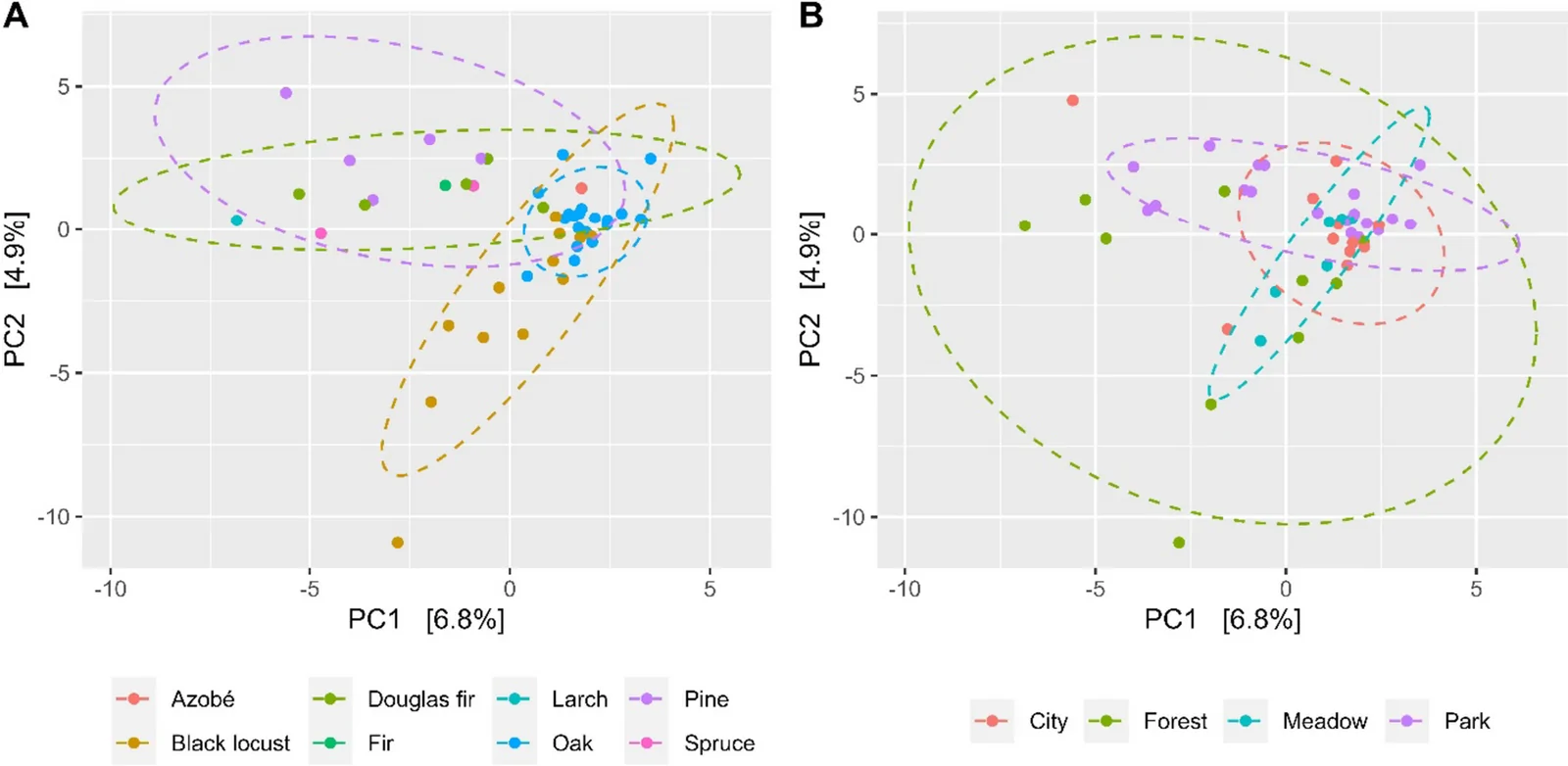
Two-dimensional PCA plots for comparing fungal community compositions based on ASV grouped **A** by wood species (*Abies alba*, *Lophira alata*, *Larix* sp., *Picea abies*, *Pseudotsuga menziesii*, *Pinus sylvestris*, *Quercus* spp., *Robinia pseudoacacia*) and **B** by its immediate environment

Multivariate analyses showed that the fungal compositions from the wood interior represent a subcommunity of the fungal community that was found on the surface areas (*ρ* < 0.001, *R*^2^ = 0.02; Supplemental Fig. S5). To focus on wood decay and what happens inside the rotten part of the wood, only data of samples taken from the inside were used for further investigating potential influences of environmental factors on the fungal community composition. Wood species significantly affected fungal composition (*ρ* < 0.001, *R*^2^ = 0.22; Fig. 2A). The close surrounding environment (city, forest, meadow, or park) influenced the fungal community composition significantly (*ρ* = 0.011, *R*^2^ = 0.08). Fungal communities of samples surrounded by parks, meadows, or residential area grouped quite well, whilst samples surrounded by forests showed less similarity to each other (Fig. 2B). No differences were observed for fungal compositions from wood pieces in or without permanent soil contact.

Differential abundance testing revealed two ASV with significantly higher abundances on samples surfaces. One was a suspected *Aureobasidium pullulans* (ASV 150) and the other a suspected *Capronia* species.(ASV 41). No further differentially abundant ASV were identified regarding the other investigated factors, i.e. wood species, soil contact, and environmental surrounding, respectively. Variance partition analyses revealed that the C content, N content, and C/N ratio did not contribute at all for explaining the variation in β-diversity (Supplemental Fig. S6).

### Bacterial community composition

Bacterial abundance, as indicated by 16S rRNA gene copy numbers, showed on average from surface material 2.6 × 10^10^ per gram wood dry weight, which was significantly higher (*ρ* < 0.001) as compared to the interior material with 1.8 × 10^9^ (Supplemental Table S3).

DNA sequencing generated from all samples of this study a total of 11.7 million reads of which 8.47 million passed quality filtering and resulted in the annotation of 12,180 ASV. After removing eukaryote-associated ASV, singletons, doubletons, and ASV that could only be classified to the domain-level, a total of 11,114 ASV remained. The majority of these belonged to *Proteobacteria* (42.1%), *Bacteroidota* (10.6%), and *Actinobacteriota* (9.6%). Other phyla with relative abundance of more than 1% were *Planctomycetota*, *Verrucomicrobiota*, *Acidobacteriota*, *Bdellovibrionota*, *Myxococcota*, *Cyanobacteria*, *Chloroflexi*, *Armatimonadota*, and *Firmicutes*. Another 25 phyla with abundance of less than 1% were also detected (Supplemental Table S6).

Regardless if obtained from surface or inside material, *Proteobacteria* had the highest relative abundance (Supplemental Fig. S7), with *Alphaproteobacteria* being the dominant subphylum comprising on average 80.6% of the relative abundance per sample. *Cyanobacteria* had a higher relative abundance in the surface samples as compared to the interior.

**Fig. 3:**
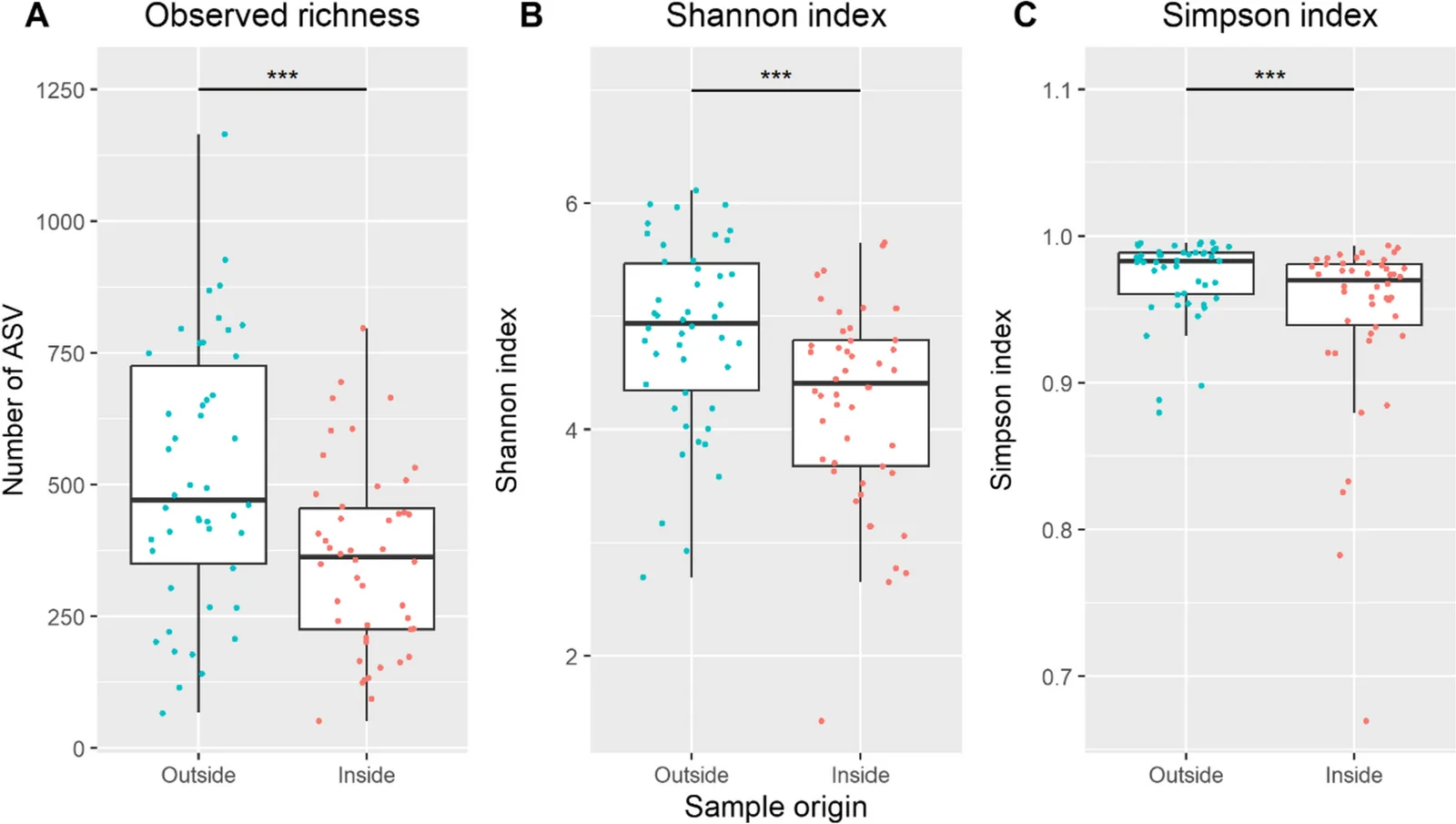
Bacterial diversity, based on 16S rRNA gene amplicon sequence variants (ASV) associated with timber wood damage, as sampled from the surface (outside) and inside material. **A** Observed richness, **B** Shannon index, and **C** Simpson index of fungal ASV between samples taken from the outside and inside. The asterisks indicate the level of statistical significance (observed: Wilcoxon signed rank test, *ρ* < 0.001; Shannon: Wilcoxon signed rank test, *ρ* < 0.001; Simpson: Wilcoxon signed rank test, *ρ* < 0.001)

Whilst in total 4060 ASV were exclusively found on the wood surface, 2654 ASV were exclusively detected on the interior, and 4400 ASV in both. Correspondingly, a higher richness (*ρ* < 0.001) was seen for wood surface samples, and accordingly, the calculated α-diversity metrics, i.e. Shannon index (*ρ* < 0.001) and Simpson index (*ρ* < 0.001), were higher compared to interior samples (Fig. 3).

**Fig. 4:**
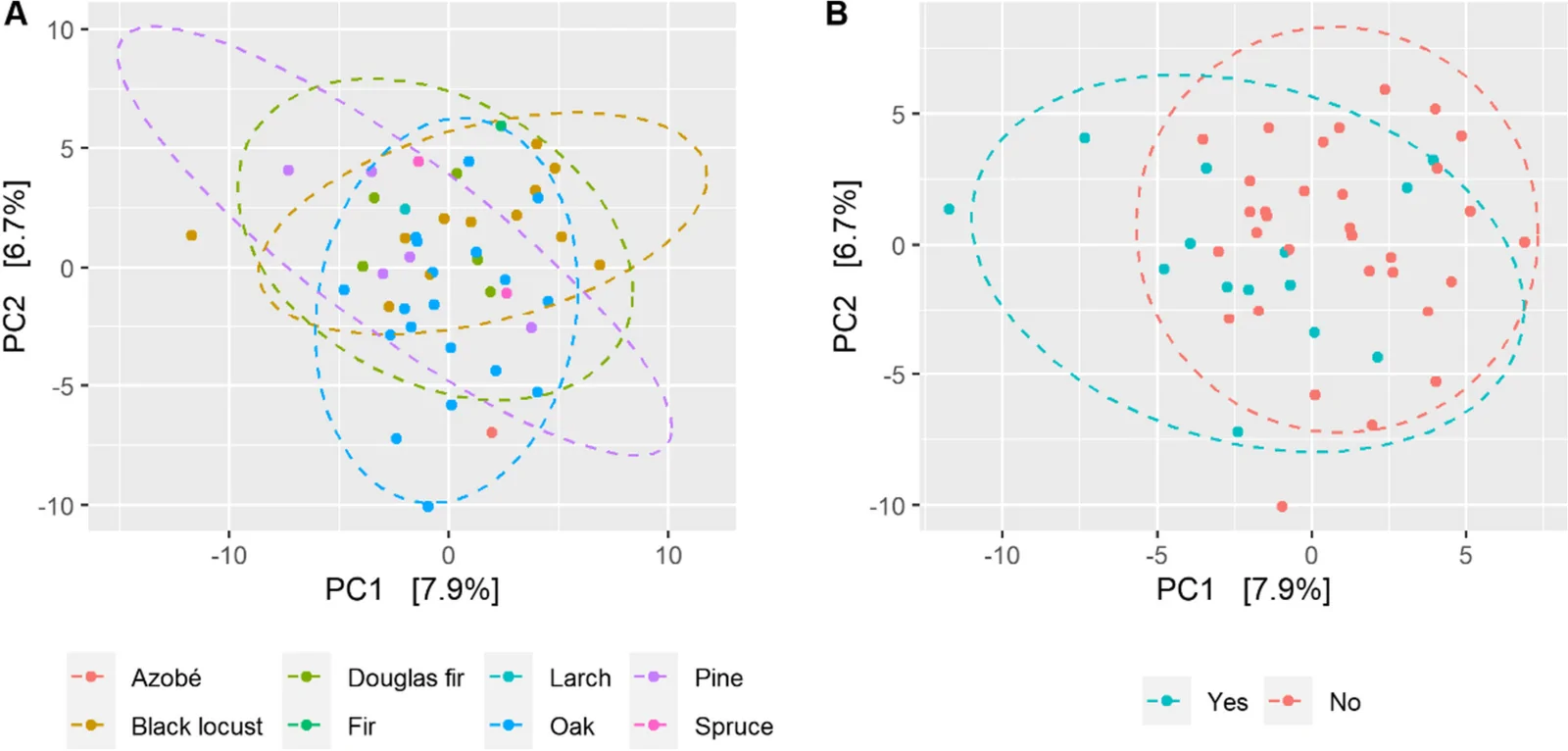
Two-dimensional PCA plot for comparing the bacterial community compositions based on ASV, grouped by **A** wood species (*Abies alba*, *Lophira alata*, *Larix* sp., *Picea abies*, *Pseudotsuga menziesii*, *Pinus sylvestris*, *Quercus* spp., *Robinia pseudoacacia*) and **B** samples with and without soil contact

The bacterial compositions of samples taken from the interior seem to represent a subcommunity of the bacterial communities found on the surface (*ρ* = 0.005, *R*^*2*^ = 0.02; Supplemental Fig. S8). The influence of environmental factors on bacterial communities was only inspected on samples taken from the inside. Wood species significantly influenced bacterial composition (*ρ* = 0.044, *R*^2^ = 0.19; Fig. 4A). In contrast to the fungal compositions, permanent soil contact of the sampled wood affected bacterial composition significantly (*ρ* = 0.017, *R*^2^ = 0.03; Fig. 4B), whilst the immediate environment had no significant effect. Additionally, bacterial composition differed significantly based on the prevalent type of wood decay (*ρ* = 0.002, *R*^2^ = 0.08; Supplemental Fig. S9).

In total four differential abundant ASV were identified between the surface and the interior of samples with all of them more abundant on the outside: two ASV (ASV 19, *ρ* = 0.003; ASV 130, *ρ* = 0.027) belonging to *Rickettsiales* (*Alphaproteobacteria*) and the other two ASV (ASV 37, *ρ* = 0.045; ASV 66, *ρ* = 0.025) belonging to *Cyanobacteria*.

Variance partition analyses showed that both C and N contents explained 0.03 of the variation in β-diversity. However, both of the factors had no significant effect (*ρ* = 0.709) on community composition (Supplemental Fig. S10).

### Network analysis

Correlation network analyses were conducted to explore potential links of fungal and bacterial taxa in response to sample types and environmental variables (Supplemental Fig. S11). In total, 582 ASV passed the quality criteria to be included in the network analyses (see the “Statistical analysis” section) and 347 ASV remain not connected to any other. In the resulting rather loose network (edge density: 0.002), a total of 64 ASV have only a single connection. The network shows some clusters formed by few ASV and one particular cluster standing out, because of its dense connectivity. This latter cluster consists of four ASV belonging to *Ascomycota* (two *Phaeoisaria*, one *Phialemoniopsis* species, one *Ophiostomatales*), six *Proteobacteria* (belonging to *Devosiaceae* two times, *Cellvibrionaceae*, *Sphingomonadaceae*, *Luteibacter*, and *R7C24* lineage), two *Actinobacteriota* (belonging to *Conexibacter* and *Micrococcales*), and two *Bacteriodota* (belonging to *Pedobacter* and *Cyclobacteriaceae*). All of these ASV were, in fact, very abundant in one single wood sample (39/40), which was the only soft rot sample. Small clusters of or single connections of dominant basidiomycetes are also detectable in the network. *Gloeophyllum sepiarium* for example is connected to four ASV belonging to *Proteobacteria* (order *Rickettsiales*) and one *Acidobacteriota* (genus *Terriglobus*). *Mycena haematopus* is linked to three ASV belonging to *Proteobacteria* (two times order *Rickettsiales* and family *Burkholderiaceae*) and one belonging to *Ascomycota* (genus *Chaetosphaeria*). The more frequently found basidiomycetes (e.g. *P. meridionalis* and *Dacrymyces* spp.) could not be associated with any other ASV. It should be noted that some clusters may not indicate co-occurrence of different taxa but simply result from ASV originating from the same taxon, e.g. ASV2, ASV81, and ASV155, all representing the basidiomycete *Xylodon raduloides*. Overall, the correlation network suggests interactions between fungi and bacteria during the colonisation and decomposition of timber wood.

## Discussion

Timber of bridges and playgrounds damaged by wood decay fungi was collected in this study from various sites within the urban area of the city of Hamburg. The basidiomycete *P. merdionalis* (*Polyporales*) was found to be frequently associated with white rot damage of oak timber. This species was only recently described after revising the genus (Decock and Stalpers 2006). This recent assignment explains the limited knowledge about its environmental occurrence and ecology. The white rot fungus was previously found to mostly colonise oak deadwood in Southern European forests and, when found in Central or Northern Europe, it mainly occurred on oak timber (Decock and Stalpers 2006; Rivoire 2020). Although its potential to degrade oak timber has apparently not yet been assessed, it has already been shown that the fungus can produce lignin-degrading enzymes and decompose lignin in alfalfa stems (Doria et al. 2014; Girometta et al. 2017). The examination of the fruiting bodies in combination with the Sanger sequencing of the ITS region showed that *P. meridionalis* was incorrectly recognised by the UNITE database as *P. vanhulleae*. The reason for the misidentification is simply that there is no reference sequence of *P. meridionalis* included in the database yet.

Three *Dacrymyces* species (*Basidiomycota*, *Dacrymycetales*) were also detected with timber wood damage and it has long been known that these fungi can cause degradation of construction timber (Seifert 1983). Their importance and frequency increased since the use of toxic additives, e.g. copper and zinc, in coatings has been reduced (Huckfeldt and Schmidt 2015). It should be noted that one of the suspected species, *D. stenosporus*, was first described in New Zealand (Shirouzu et al. 2017), and there are yet no findings recorded for Germany. Considering the geographical distance, it seems possible that the taxon detected in this study with wood damage originated from a native *Dacrymyces* that is not included in the UNITE database and not yet characterised. The taxonomic resolution of the genetic marker used in this study, i.e., the ITS2 region, does not allow a stand-alone unambiguous taxonomic assignment (Badotti et al. 2017; Stadler et al. 2020).

Wood species was in this study the analysed factor that explained the largest proportion of variation of fungal community composition, as revealed by multivariate statistics. This is in line with results from numerous deadwood studies which demonstrated the importance of tree species for the fungal community composition (Baldrian et al. 2016; Lepinay et al. 2022). Wood-inhabiting fungi can have strong host preferences and form distinctive species-specific communities (Purahong et al. 2017). These wood-specific preferences can be partially explained by different physico-chemical wood properties, i.e. pH, the amount of C and N, and their ratio. Especially, the pH value is known to have a strong influence on the fungal decomposition of wood (Lepinay et al. 2022; Purahong et al. 2018), as fungal growth is generally better in a slightly acidic environment (Rousk and Bååth 2011). Wood degrading fungi even acidify their surroundings to facilitate their access to nutrients. White rot fungi produce a complete enzymatic system enabling them to degrade all major wood components (e.g. cellulose, hemicellulose, and lignin; Goodell et al. 2008). Brown rot fungi, on the other hand, use a combination of hydrolytic enzymes and oxidative low molecular weight compounds (e.g. oxalic acid) to degrade cellulose and hemicellulose whilst lignin only gets slightly altered (Arantes and Goodell 2014). They can also use lytic polysaccharide monooxygenases (LPMOs) to break down cellulose (Kojima et al. 2016). Although wood is acidified in both cases, wood affected by brown rot fungi is significantly more acidic (Goodell et al. 2002). In case of C and N content, as well as C/N ratio, it was shown that they can be good indicators for fungal community composition, when comparing the communities of two phylogenetically distant tree species (coniferous vs deciduous; Hoppe et al. 2016; Lepinay et al. 2021), but not when comparing more or phylogenetically closer related species (Purahong et al. 2018). This might also explain why no influence of chemical parameters was found in this study.

The immediate surrounding environment was another important factor influencing the fungal community composition. The PCA plot revealed that fungal communities of rotten wood surrounded by forests did not group very well in comparison to other environments, suggesting that they may have distinct effects depending on other factors, e.g. type of forest or their location within the landscape. But most of all, deadwood availability could be an important factor promoting fungal diversity (Bässler et al. 2010). A lack of colonisable wood in urban environments has been reported (Fröhlich and Ciach 2020). Dead wood in the surrounding forests could serve a reservoir for a greater diversity of wood decomposing fungi, from which spores can escape over time. This explanation is supported by the fact that fungal species richness is higher in near-natural landscapes than in urban environments (Englmeier et al. 2023).

The bacterial community composition detected in this study with timber wood damage was dominated by *Proteobacteria*, *Actinobacteriota*, *Bacteroidota*, and *Acidobacteriota*. These dominant phyla and their proportion to each other were similar to communities found in deadwood (Moll et al. 2018; Tláskal et al. 2017), and in fact not much different from communities as they typically occur in soil or litter (Labouyrie et al. 2023; Lladó et al. 2017). It was reported that around 10% of litter bacteria are able to degrade cellulose, of which the majority belonged to *Proteobacteria*, *Actinobacteriota*, *Bacteroidota*, and *Acidobacteriota* (Lladó et al. 2017; López-Mondéjar et al. 2016). As these were also the dominating phyla colonising timber wood in our study, it is likely that they also take part in the cellulolytic decomposition of timber. It was shown that bacterial communities in early deadwood stages are dominated by bacteria with an increased potential to utilise complex polysaccharides like cellulose, which are then replaced in later decay stages by opportunistic bacteria, mostly belonging to *Alphaproteobacteria* and *Gammaproteobacteria*, the latter suspected to rely on by-products from fungal degradation or mycophagy (Tláskal and Baldrian 2021).

Similar to fungal communities, wood species was also for bacterial communities the most important factor shaping their composition, and this has equally been observed with deadwood in other studies (Hoppe et al. 2015; Moll et al. 2018). One explanation for these wood species depending differences could again be their characteristic physico-chemical wood properties. Especially pH was repeatedly reported as a main driver of bacterial community composition (Johnston et al. 2019; Tláskal et al. 2017). Wood with a high pH (e.g., *Tilia* sp., *Fraxinus* sp., or *Populus* sp.) was colonised by different bacterial communities as compared to wood with lower pH values like *Pinus* or *Picea* (Moll et al. 2018). Furthermore, wood-decaying fungi decrease the wood pH significantly during the decomposition process, which than will affect the bacterial community composition by shifting it towards a more acido-tolerant community (Boer et al. 2010; Haq et al. 2022). This acidification process is generally more pronounced with brown rot than white rot, and this may explain the differences in bacterial community composition based on prevalent rot type in our data set (Supplemental Fig. S9). Unfortunately, the pH of the damaged timber wood of the samples collected in this study could not be measured due to limited amount of material that we were allowed to sample. For the bacterial community composition, other studies demonstrated the importance of the wood decay stage, which outweighed the differences found between different wood species (Müller et al. 2020; Tláskal et al. 2017). This influence could not be assessed in this study because repeated sampling of the same damaged timber wood was not allowed by the local authorities.

The bacterial community composition in samples with soil contact differed clearly in this study from samples without soil contact. Soil provides a habitat for many bacteria with the capacity to degrade organic polymeric substances, and thus, it is likely that this contact strongly facilitated access of such bacteria to colonise the open niche of timber with celluloses and lignin as carbon and energy sources. Probably because of the limited number of samples and their natural variability in the bacterial community composition, it was unfortunately not possible to detect particular taxa (ASV) with significantly differential abundance between timber wood with and without soil contact. Thus, the sampling design did not allow univocal conclusions on the relationship between ASV and wood species and the difference between environments from which they were collected. However, our data demonstrate variation of microbial communities in outdoor timber wood constructions within the same regional area. To identify early microbial indicators of timber wood decomposition and explore new means of controlling them more extensive sampling campaigns would be needed, for which support of the local authorities would be fundamental.

In conclusion, our study demonstrates that both fungal and bacterial communities of timber are generally similar to those inhabiting deadwoods. This is probably rooted in the fact that wood species was the most important factor shaping both fungal and bacterial community composition. Whilst for the fungal community composition the immediate environment was important, a major influence on the bacterial community composition of timber with soil contact. Our data suggest that the basidiomycete *P. meridionalis* may play a greater role in the decomposition of oak wood structures than yet acknowledged. More extensive sampling surveys are needed to better assess the possible impact of this fungus on timber wood decomposition. A better understanding of the biogeography of fungal and bacterial communities associated with biodegradation of timber wood at regional scales and beyond could contribute to early indication of damages and support the development of strategies for an environmentally friendly control.

## Supplementary Information

Below is the link to the electronic supplementary material.

Supplementary file1 (PDF 1684 KB)

## Data Availability

Data of this study are available at OA (OpenAgrar; www.openagrar.de; https://doi.org/10.3220/DATA20240219102640-0). DNA sequences of this study can be found at the European Nucleotide Archive under Project Accession Numbers PRJEB63071 and OY979657 to OY979661, respectively.
