# Supplementary material for: Identifying environmental factors affecting the microbial community composition on outdoor structural timber
Source: Appl Microbiol Biotechnol. 2024 Mar 6;108:254. doi: 10.1007/s00253-024-13089-3 (PMC10917859; doi:10.1007/s00253-024-13089-3)
Supplement: Supplementary file 1 [file 253_2024_13089_MOESM1_ESM.pdf]

Supplementary Materials

Applied Microbiology and Biotechnology

Identifying environmental factors affecting the microbial community composition on outdoor structural timber

Lauritz Schrader[a], Jochen Trautner[a], Christoph C. Tebbe[b*]

[a]Thünen Institute of Wood Research, Leuschnerstraße 91, 21031 Hamburg, Germany

[b]Thünen Institute of Biodiversity, Bundesallee 65, 38116 Braunschweig, Germany

[*]corresponding author: E-Mail address: christoph.tebbe@thuenen.de

Contents:

6 Tables and 11 Figures

Table S1 Summary of all testvariables for each wood sample (combined surface and inside samples), C and N content was measured only for samples from the interior.

Sample-ID	Wood species	Type of decay	Soil contact	Immediate environment	N [%]	C [%]	C/N ratio
01 / 02	*Pinus sylvestris*	Brown rot	Yes	Park	0.19	52.28	275
03 / 04	*Pinus sylvestris*	Brown rot	No	Park	0.33	50.55	153
05 / 06	*Pseudotsuga menziesii*	Brown rot	No	Forest	0.18	50.67	282
07 / 08	*Quercus* sp.	White rot	Yes	City	0.25	48.01	192
09 / 10	*Quercus* sp.	White rot	No	Park	0.37	49.05	132
11 / 12	*Quercus* sp.	White rot	Yes	Park	0.34	48.39	141
13 / 14	*Pseudotsuga menziesii*	Brown rot	Yes	Park	0.2	49.76	245
15 / 16	*Picea abies*	Brown rot	No	Park	0.37	52.31	141
17 / 18	*Robinia pseudoacacia*	Brown rot	Yes	Meadow	0.43	48.71	113
19 / 20	*Quercus* sp.	White rot	No	Meadow	0.56	48.32	86
21 / 22	*Quercus* sp.	White rot	Yes	Meadow	0.37	37.4	102
23 / 24	*Robinia pseudoacacia*	Brown rot	No	Meadow	0.32	49.73	155
25 / 26	*Quercus* sp.	White rot	No	Park	0.38	49.42	131
27 / 28	*Quercus* sp.	White rot	No	Park	0.47	48.68	103
29 / 30	*Robinia pseudoacacia*	White rot	Yes	Meadow	0.28	49.82	177

31 / 32	*Robinia pseudoacacia*	Brown rot	No	Meadow	0.25	49.32	196
33 / 34	*Quercus* sp.	White rot	No	Forest	0.47	48.58	103
35 / 36	*Abies alba*	Brown rot	No	Forest	0.16	45.8	286
37 / 38	*Robinia pseudoacacia*	White rot	No	Forest	0.56	49.19	88
39 / 40	*Robinia pseudoacacia*	Soft rot	Yes	Forest	0.37	49.41	133
43 / 44	*Picea abies*	Brown rot	No	Forest	0.2	51.51	255
45 / 46	*Larix* sp.	Brown rot	No	Forest	0.29	50.13	173
47 / 48	*Robinia pseudoacacia*	White rot	Yes	City	0.35	48.9	142
49 / 50	*Robinia pseudoacacia*	White rot	No	City	0.27	49.37	181
51 / 52	*Robinia pseudoacacia*	Brown rot	No	City	0.29	49.78	172
53 / 54	*Lophira alata*	White rot	No	Park	0.33	50.92	154
55 / 56	*Pseudotsuga menziesii*	White rot	No	Park	0.32	49.63	155
57 / 58	*Quercus* sp.	Brown rot	No	Park	0.7	49.15	70
59 / 60	*Quercus* sp.	White rot	No	Park	0.38	48.83	129
63 / 64	*Pseudotsuga menziesii*	Brown rot	Yes	Park	0.18	50.34	280
65 / 66	*Quercus* sp.	White rot	Yes	Park	0.21	49.63	233
67 / 68	*Pinus sylvestris*	Brown rot	No	City	0.34	52.7	155
69 / 70	*Quercus* sp.	White rot	No	City	0.51	48.25	94
71 / 72	*Quercus* sp.	Brown rot	No	City	0.34	49.61	146
73 / 74	*Quercus* sp.	White rot	No	City	0.27	49.09	185
75 / 76	*Pinus sylvestris*	Brown rot	No	Park	-	-	-
77 / 78	*Pseudotsuga menziesii*	Brown rot	No	Park	0.32	51.1	159
79 / 80	*Pinus sylvestris*	Brown rot	No	Park	0.14	52.22	367
81 / 82	*Quercus* sp.	White rot	No	City	0.21	49.12	230
83 / 84	*Quercus* sp.	White rot	No	City	0.38	49.1	129
85 / 86	*Quercus* sp.	Brown rot	Yes	City	0.31	49.89	163
87 / 88	*Robinia pseudoacacia*	Brown rot	Yes	Forest	0.29	49.41	171
89 / 90	*Robinia pseudoacacia*	Brown rot	No	Forest	0.26	49.75	191
91 / 92	*Robinia pseudoacacia*	White rot	No	Forest	0.29	49.25	170
93 / 94	*Quercus* sp.	White rot	Yes	Park	0.28	49.75	181
95 / 96	*Quercus* sp.	White rot	No	Park	0.47	47.72	101

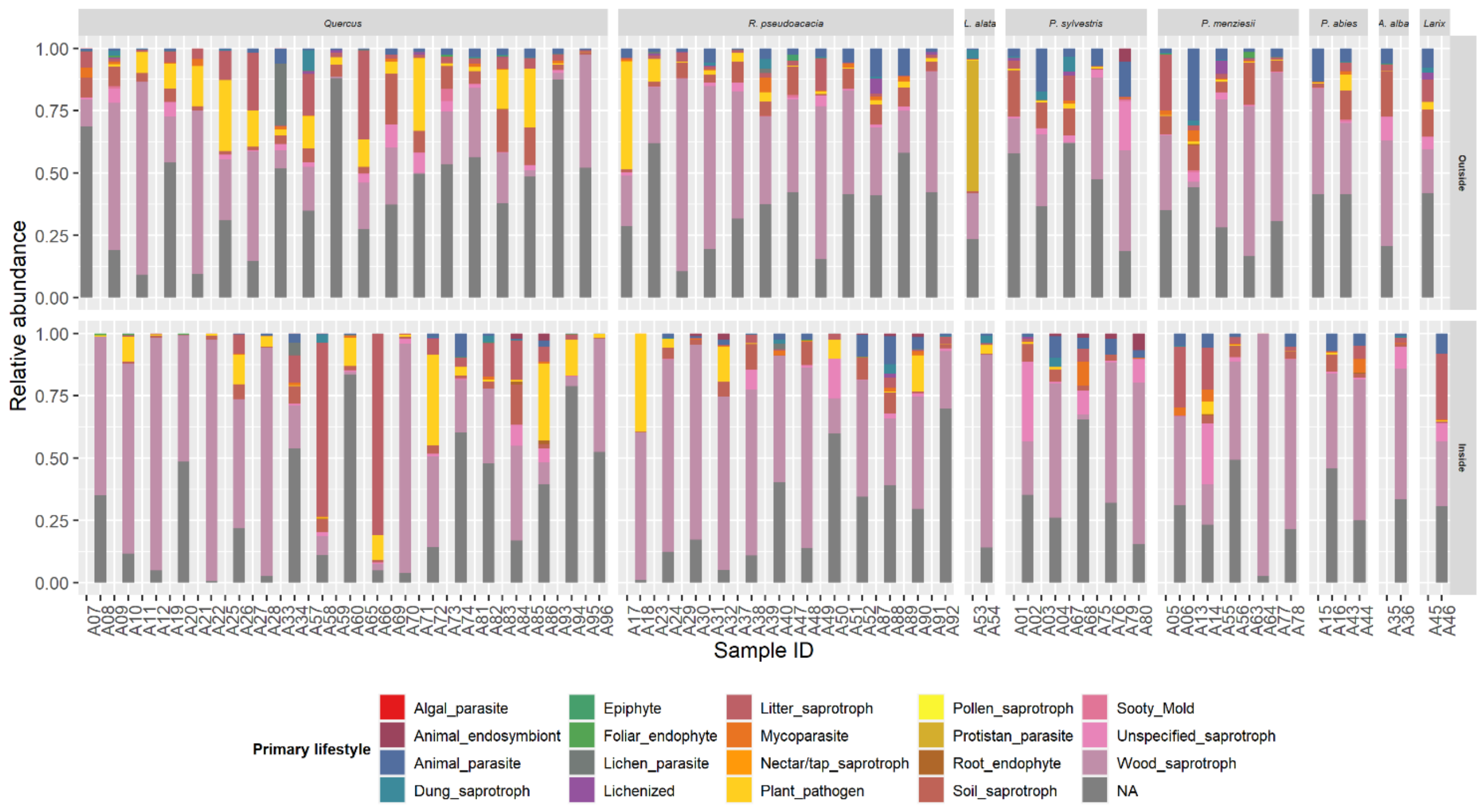

Figure S1 Suspected primary lifestyles of fungal ASVs structred by sample origin and wood species

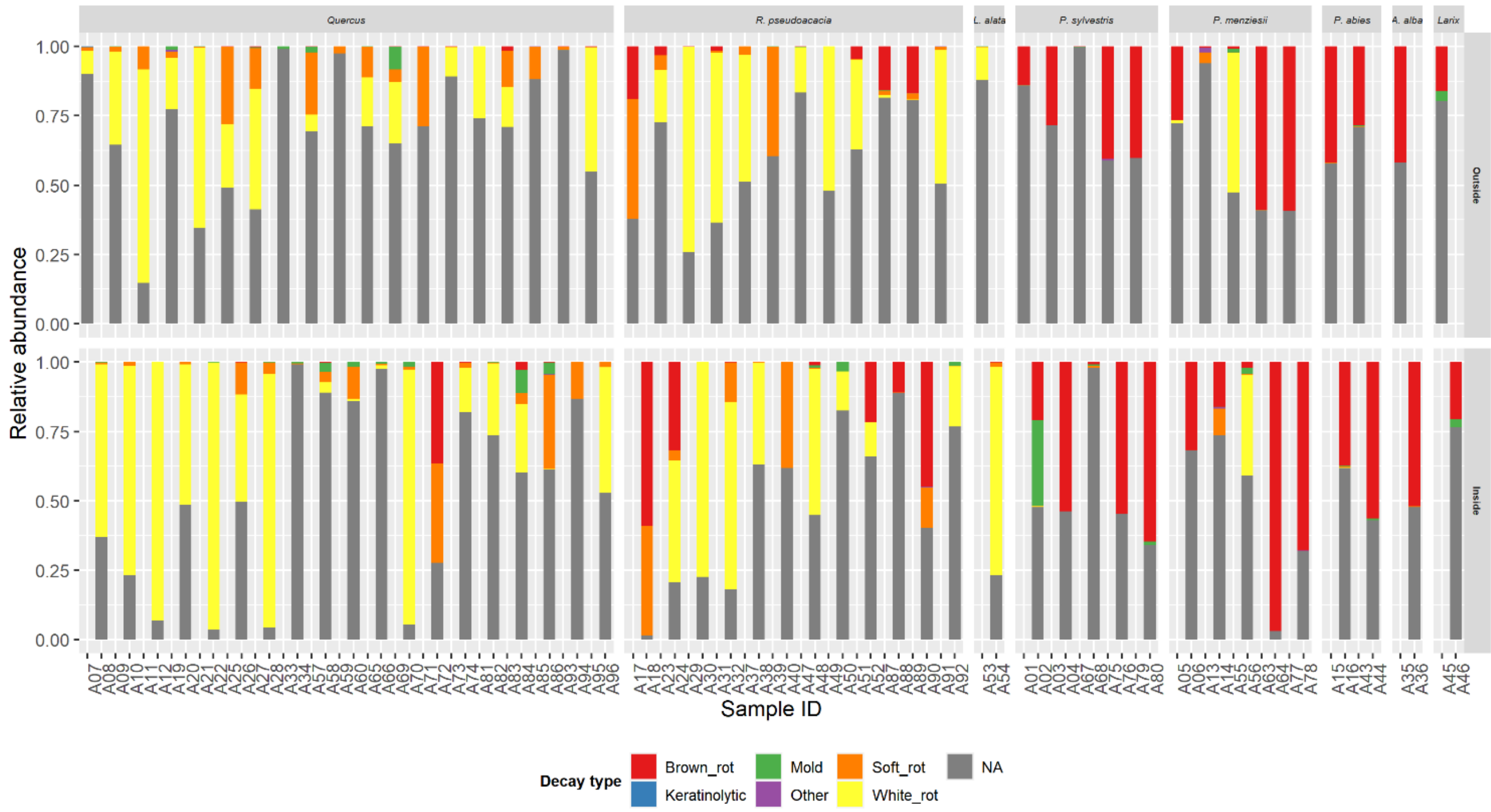

Figure S2 Assignment of fungal ASV according to the rot type they are typically associated with, as detected with different wood species

30

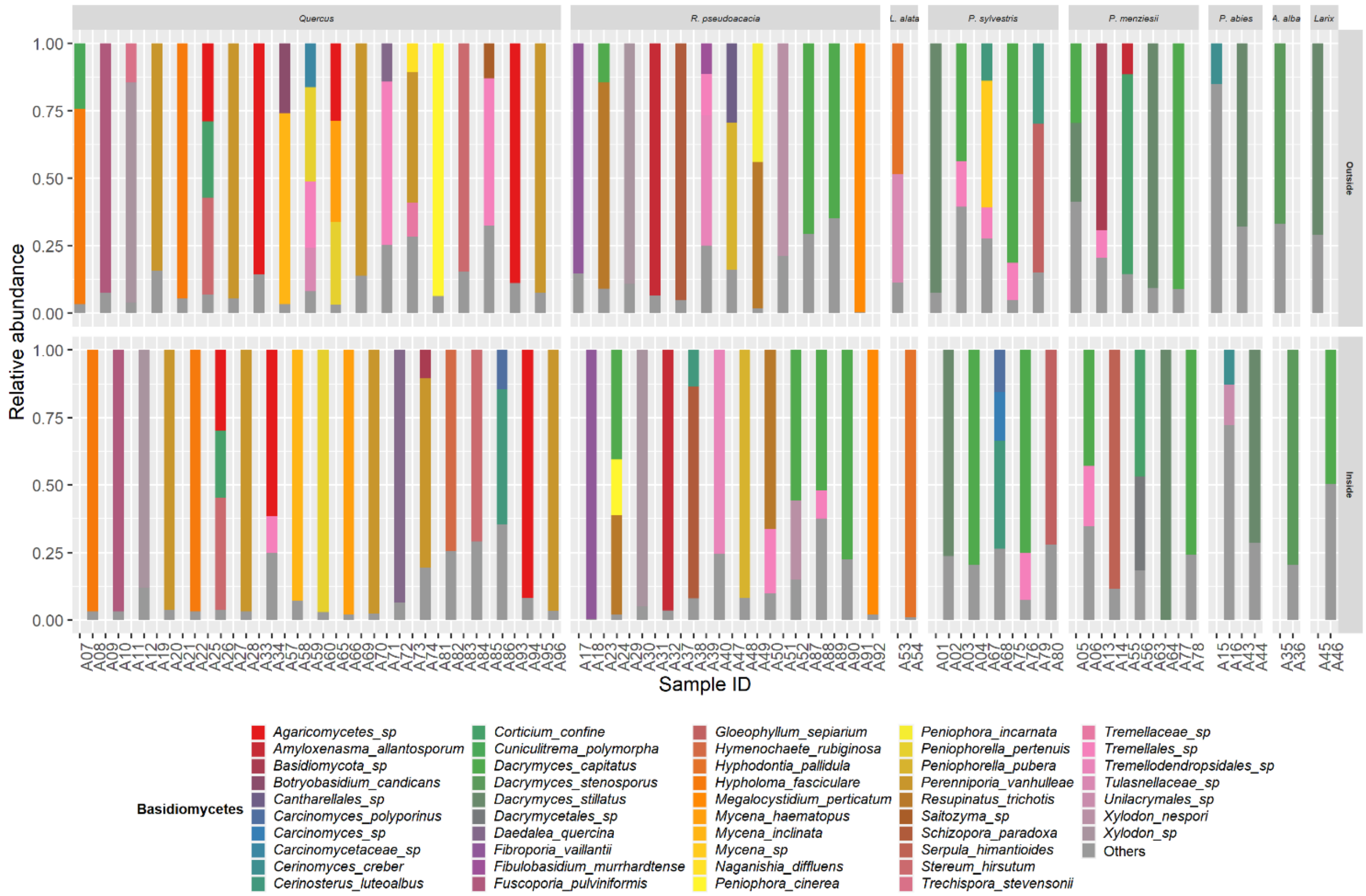

Figure S3 Relative abundance of fungal ASV assigned to the phylum *Basidiomycota*. Only ASV with at least 10% relative abundance are indicated and grouped according to the wood species where they were detected. Species/Taxa with < 10 % are are grouped as „Others“

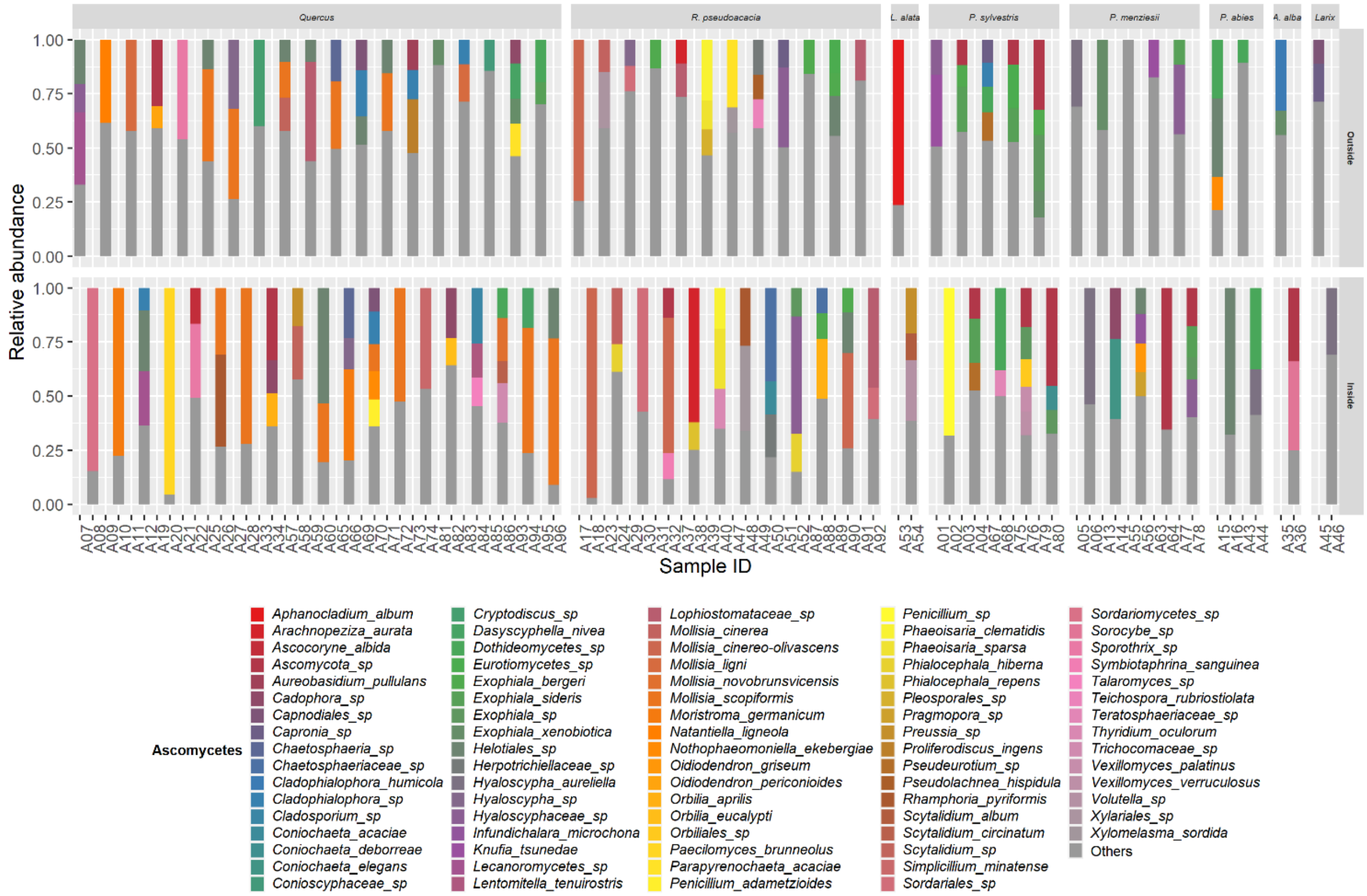

Figure S4 Fungal ASV affiliated with *Ascomycota* with at least 10% relative abundance grouped according ot the wood type from where they were detected. Species/Taxa with < 10 % are are grouped as „Others“

Table S2 Wood chemical properties, i. e. C and N contents, and C : N ratio of wood samples as detected with the different wood species and, where possible the respective decay type. Standard errors were calculated for all combinations with at least n=2.

Woodspecies	Decay type	C content [%]	N content [%]	C/N ratio
Quercus spp.	White rot	48.1 ± 0.71	0.37 ± 0.03	142 ± 12
	Brown rot	49.6 ± 0.26	0.45 ± 0.15	126 ± 35
Robinia pseudoacacia	White rot	49.3 ± 0.14	0.35 ± 0.05	151 ± 16
	Brown rot	49.4 ± 0.18	0.30 ± 0.03	166 ± 13
Lophira alata	White rot	50.9	0.33	154
Abies alba	Brown rot	45.8	0.16	286

Larix sp.	Brown rot	50.1	0.29	172
Picea abies	Brown rot	51.9 ± 0.40	0.29 ± 0.09	198 ± 57
Pinus sylvestris	Brown rot	51.9 ± 0.48	0.25 ± 0.05	238 ± 52
Pseudotsuga menziesii	White rot	49.6	0.32	155
	Brown rot	50.5 ± 0.28	0.22 ± 0.03	241 ± 29

Table S3 Abundance of fungal ITS2 and bacterial 16S rRNA genes as indicators for their biomass as detected by qPCR from the different timber wood samples (copy numbers per g wood dry weight). Odd sample numbers indicate surface samples, even numbers indicate samples from the interior timber damages.

Sample-ID	ITS2	16S rRNA genes
1	2.99 x 10^{10}	4.50 x 10^{10}
2	3.12 x 10^{7}	1.37 x 10^{7}
3	1.12 x 10^{10}	6.01 x 10^{9}
4	1.50 x 10^{10}	2.56 x 10^{9}
5	4.26 x 10^{10}	1.62 x 10^{10}
6	8.02 x 10^{9}	2.54 x 10^{9}
7	6.52 x 10^{9}	1.38 x 10^{10}
8	2.20 x 10^{8}	1.02 x 10^{6}
9	6.36 x 10^{7}	3.14 x 10^{7}
10	1.84 x 10^{10}	2.24 x 10^{8}
11	5.83 x 10^{11}	1.48 x 10^{10}
12	7.60 x 10^{11}	9.78 x 10^{9}
13	3.85 x 10^{10}	5.02 x 10^{10}
14	6.09 x 10^{9}	7.91 x 10^{8}
15	8.85 x 10^{9}	1.05 x 10^{10}
16	4.45 x 10^{9}	5.43 x 10^{9}
17	1.37 x 10^{11}	5.23 x 10^{10}
18	2.64 x 10^{9}	1.85 x 10^{8}
19	1.28 x 10^{10}	6.53 x 10^{8}
20	1.20 x 10^{10}	7.61 x 10^{5}
21	1.32 x 10^{11}	2.60 x 10^{10}
22	7.93 x 10^{9}	6.98 x 10^{7}
23	3.88 x 10^{11}	5.60 x 10^{10}
24	9.46 x 10^{9}	4.47 x 10^{8}
25	2.64 x 10^{11}	5.39 x 10^{10}
26	4.71 x 10^{10}	4.16 x 10^{9}
27	1.41 x 10^{11}	2.48 x 10^{10}

28	2.62×10^{10}	1.09×10^{8}
29	5.16×10^{11}	5.44×10^{10}
30	7.31×10^{10}	4.49×10^{9}
31	2.49×10^{11}	2.91×10^{10}
32	7.81×10^{9}	2.00×10^{8}
33	9.85×10^{10}	6.48×10^{9}
34	5.01×10^{9}	1.74×10^{9}
35	3.90×10^{10}	1.18×10^{10}
36	4.60×10^{9}	1.44×10^{9}
37	2.42×10^{11}	2.75×10^{10}
38	1.56×10^{10}	5.22×10^{9}
39	4.57×10^{10}	9.32×10^{10}
40	6.99×10^{9}	8.67×10^{9}
43	1.86×10^{10}	2.09×10^{10}
44	1.73×10^{10}	1.85×10^{9}
45	7.42×10^{10}	4.17×10^{10}
46	9.12×10^{9}	2.37×10^{9}
47	1.99×10^{10}	2.01×10^{10}
48	1.78×10^{10}	1.19×10^{9}
49	2.52×10^{11}	4.92×10^{10}
50	1.93×10^{8}	1.06×10^{7}
51	5.39×10^{10}	1.21×10^{10}
52	4.59×10^{9}	1.04×10^{9}
53	2.24×10^{10}	5.40×10^{10}
54	6.57×10^{9}	2.01×10^{9}
55	1.71×10^{11}	1.10×10^{10}
56	2.17×10^{10}	9.41×10^{8}
57	2.18×10^{9}	2.98×10^{9}
58	3.90×10^{10}	4.92×10^{9}
59	5.04×10^{10}	5.61×10^{9}
60	5.40×10^{10}	4.77×10^{9}
63	4.38×10^{10}	5.10×10^{9}
64	3.91×10^{10}	8.84×10^{7}
65	8.51×10^{10}	1.68×10^{10}
66	1.18×10^{10}	9.22×10^{7}
67	2.06×10^{10}	1.58×10^{10}
68	1.00×10^{10}	2.63×10^{9}
69	2.44×10^{10}	4.70×10^{9}
70	5.94×10^{9}	4.72×10^{6}
71	1.13×10^{11}	3.37×10^{10}

72	6.55 x 10^{9}	1.94 x 10^{9}
73	4.66 x 10^{9}	6.00 x 10^{8}
74	1.47 x 10^{10}	2.79 x 10^{8}
75	1.96 x 10^{10}	5.30 x 10^{9}
76	3.93 x 10^{9}	3.60 x 10^{8}
77	1.57 x 10^{11}	1.79 x 10^{10}
78	1.42 x 10^{10}	2.36 x 10^{9}
79	2.15 x 10^{10}	2.97 x 10^{9}
80	1.10 x 10^{10}	1.22 x 10^{8}
81	2.10 x 10^{11}	3.55 x 10^{10}
82	2.15 x 10^{9}	2.07 x 10^{8}
83	1.72 x 10^{11}	4.43 x 10^{10}
84	5.33 x 10^{9}	2.28 x 10^{9}
85	1.23 x 10^{11}	7.67 x 10^{10}
86	1.25 x 10^{9}	2.92 x 10^{8}
87	4.94 x 10^{10}	1.33 x 10^{10}
88	4.96 x 10^{9}	1.28 x 10^{9}
89	2.28 x 10^{10}	7.77 x 10^{9}
90	1.71 x 10^{9}	8.03 x 10^{7}
91	3.69 x 10^{11}	7.06 x 10^{10}
92	9.41 x 10^{9}	3.20 x 10^{9}
93	5.98 x 10^{10}	1.57 x 10^{10}
94	2.88 x 10^{9}	4.00 x 10^{7}
95	3.57 x 10^{11}	7.11 x 10^{9}
96	7.61 x 10^{10}	1.80 x 10^{8}

Table S4 Number of fungal ASV divided by Division after removing single- and doubletons

**Division**	**Number of ASV**	**Proportion [%]**
Ascomycota	2,266	64.1
Basidiomycota	661	18.7
Fungi phy incertae sedis	65	1.8
Mucoromycota	28	0.79
Rozellomycota	23	0.65
Chytridiomycota	4	0.11

Olpidiomycota	2	0.06
Unidentified	487	13.8

Table S5 Characteristics of basidiospores (n = 25) from five different fruitbodies and percent identity shared with *Perenniporia meridionalis* using NCBI Blast. See Table S1 for more information on Sample IDs

Sample-ID	Basidiospores (mean size) [µm]	Dextrinoid reaction (spores)	Cyanophilic reaction (spores)	Percent identity with *P. merdionalis*
19 / 20	7.1 × 5.5	slight - strong	Yes	99.4
59 / 60	6.8 × 5.4	slight - strong	Yes	99.8
69 / 70	6.7 × 5.1	slight - strong	Yes	99.3
73 / 74	6.7 × 5.6	slight - strong	Yes	99.2
95 / 96	7.0 × 5.4	slight - strong	Yes	99.4

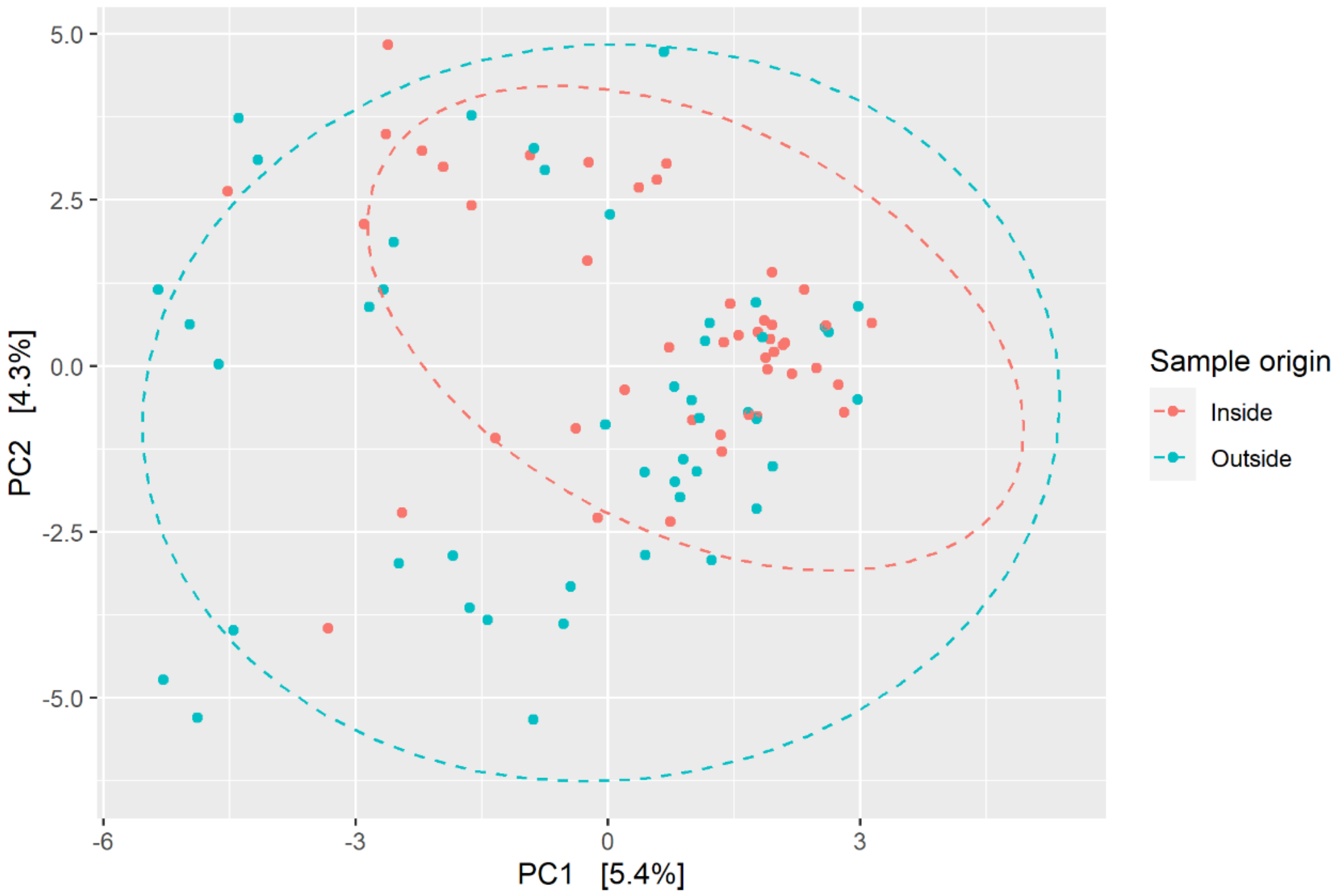

Figure S5 Two-dimensional PCA-Plot to visualize the compositiona differences of fungal communities as affected by the sample origin, either collected from surface material ("outside") or within damaged wood

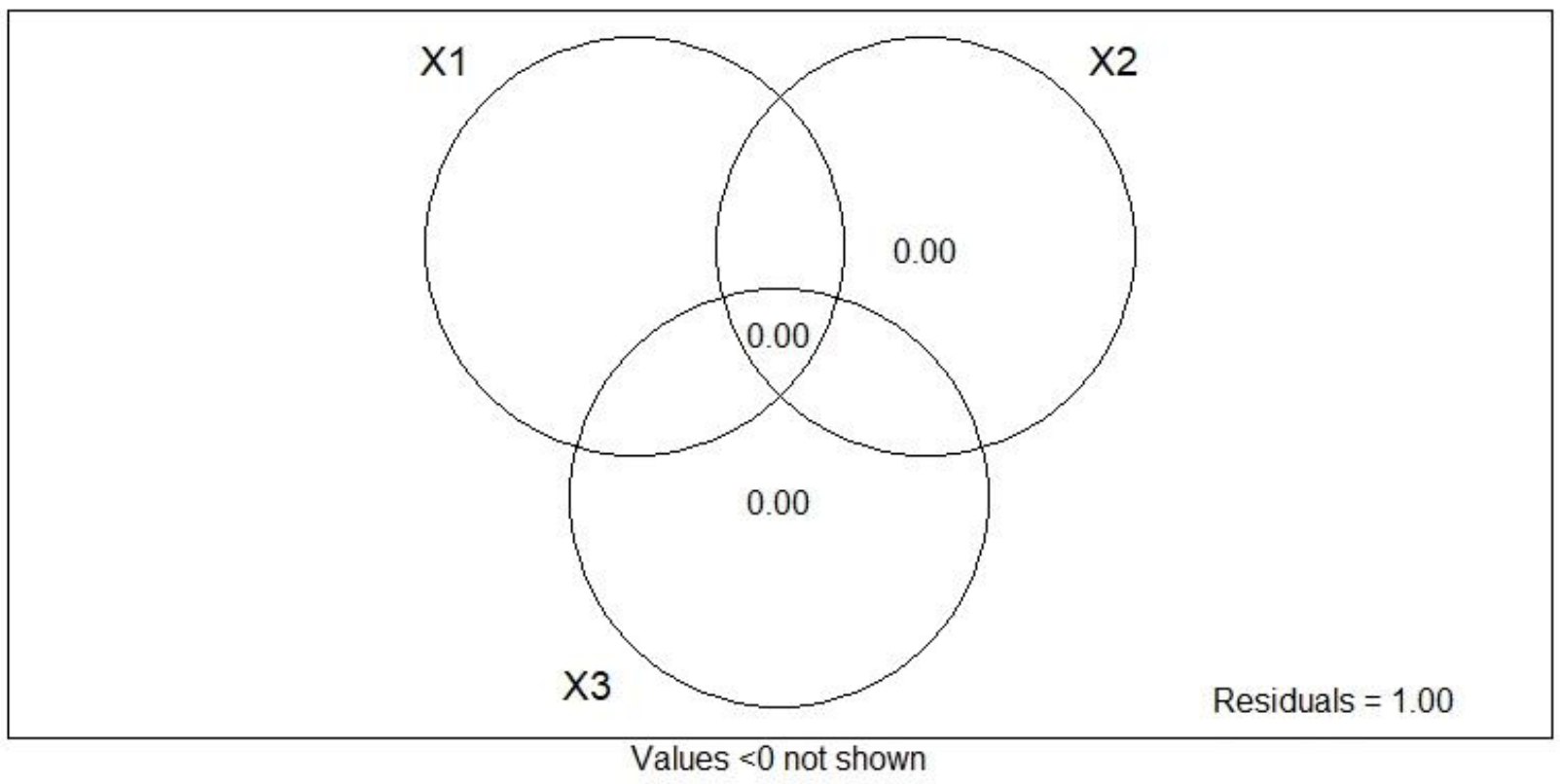

Figure S6 Variation partition to analyze the effect of total carbon (C), nitrogen (N) and the C/N ratio on the fungal community composition (X1 = C, X2 = N, X3 = C/N ratio)

Table S6 Number of bacterial ASV assinged to the different phyla, after removing single- and doubleton sequences

**Phylum**	**Number of ASV**	**Proportion [%]**
Proteobacteria	4,683	42.1
Bacteriodota	1,180	10.6
Actinobacteriota	1,067	9.60
Planctomycetota	960	8.64
Verrucomicrobiota	752	6.77
Acidobacteriota	745	6.70
Bdellovibrionta	255	2.29
Myxococcota	227	2.04
Cyanobycteria	226	2.03
Chloroflexi	211	1.90
Armatimonadota	192	1.73
Firmicutes	175	1.58
Abditibacteriota	78	0.70
Gemmatimonadota	67	0.60
WPS-2	46	0.41
Others	250	2.25

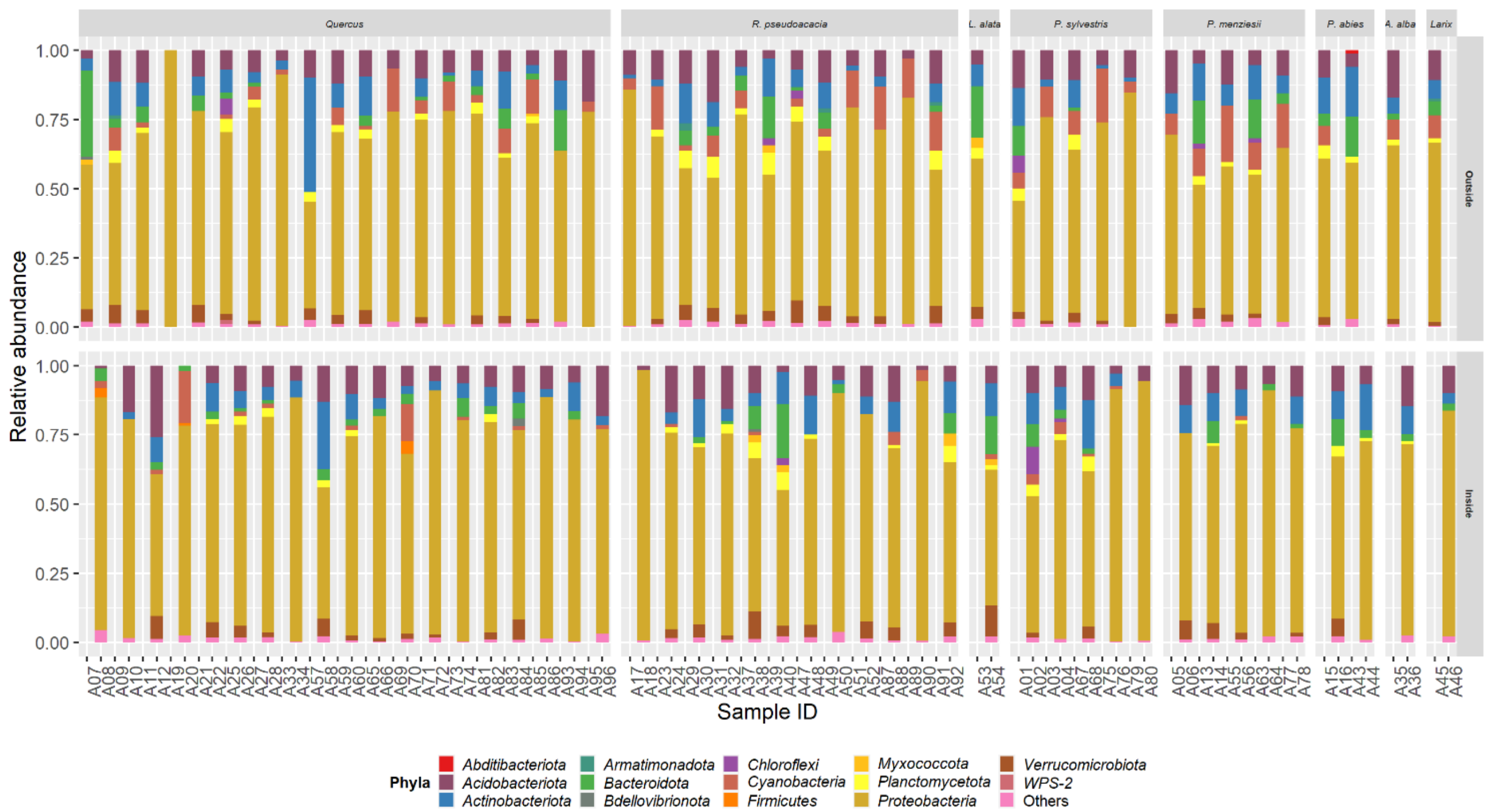

Figure S7 Bacterial Phyla with > 1% rel. abundance per sample, grouped according to wood species from which the were obtained. Phyla with < 1 % are are grouped as „Others“

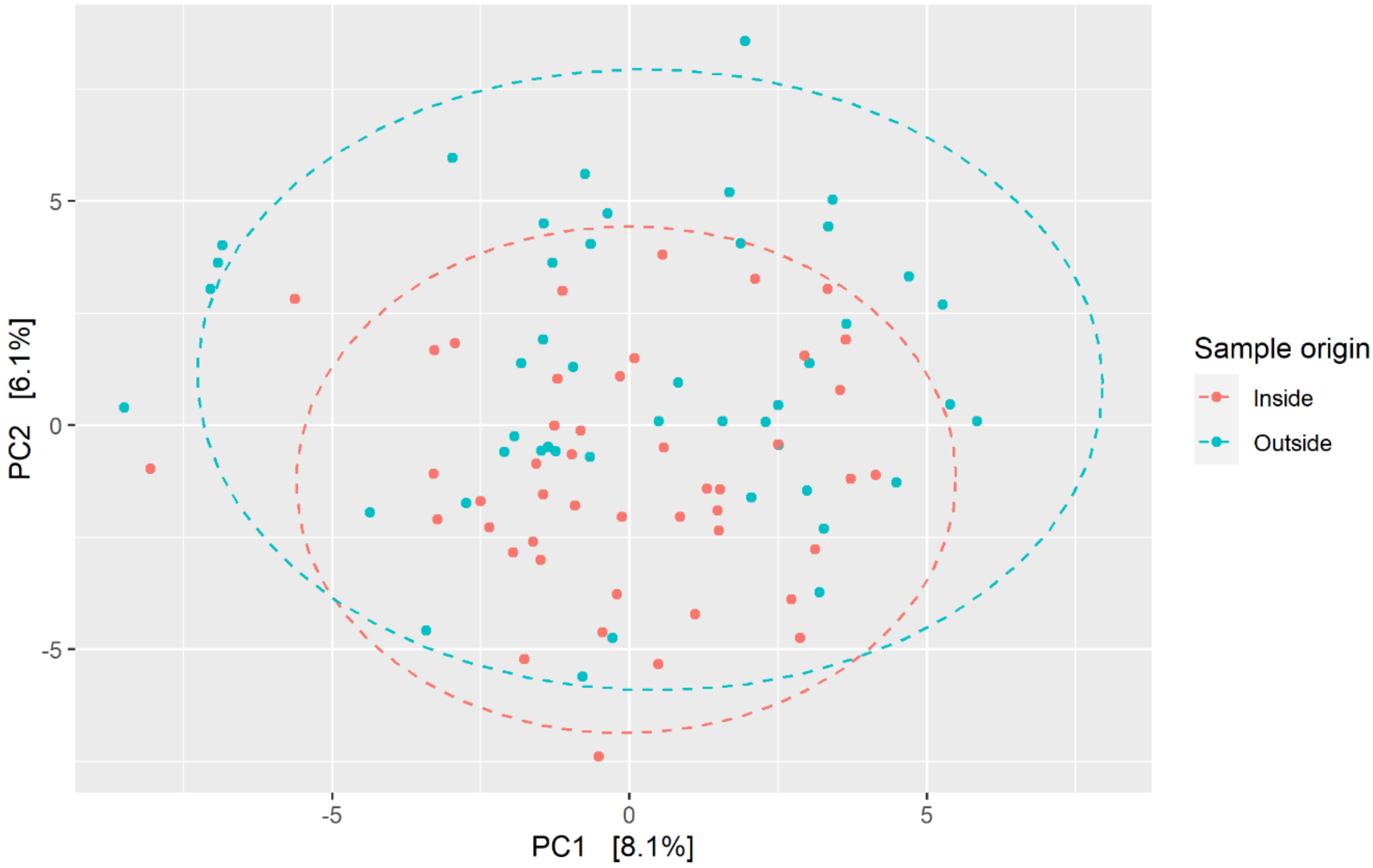

85 **Figure S8** Two-dimensional PCA-Plot to analyze the effect of collecting samples from the surface (“outside”) and the inside of damaged wood material on the bacterial communitiy composition

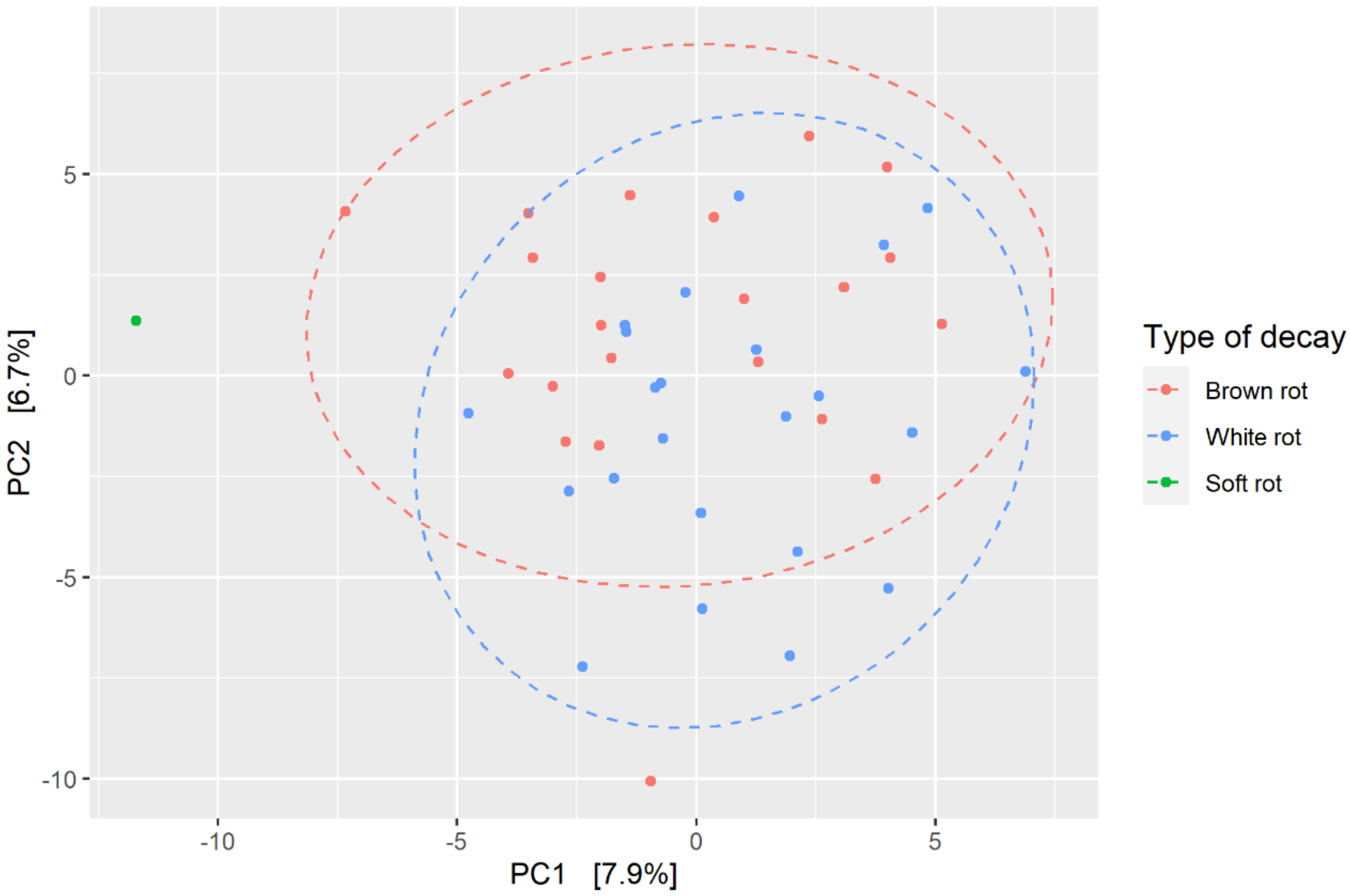

Figure S9 Two-dimensional PCA-Plot to analyze the effect of the rot type on the bacterial communitiy compositions

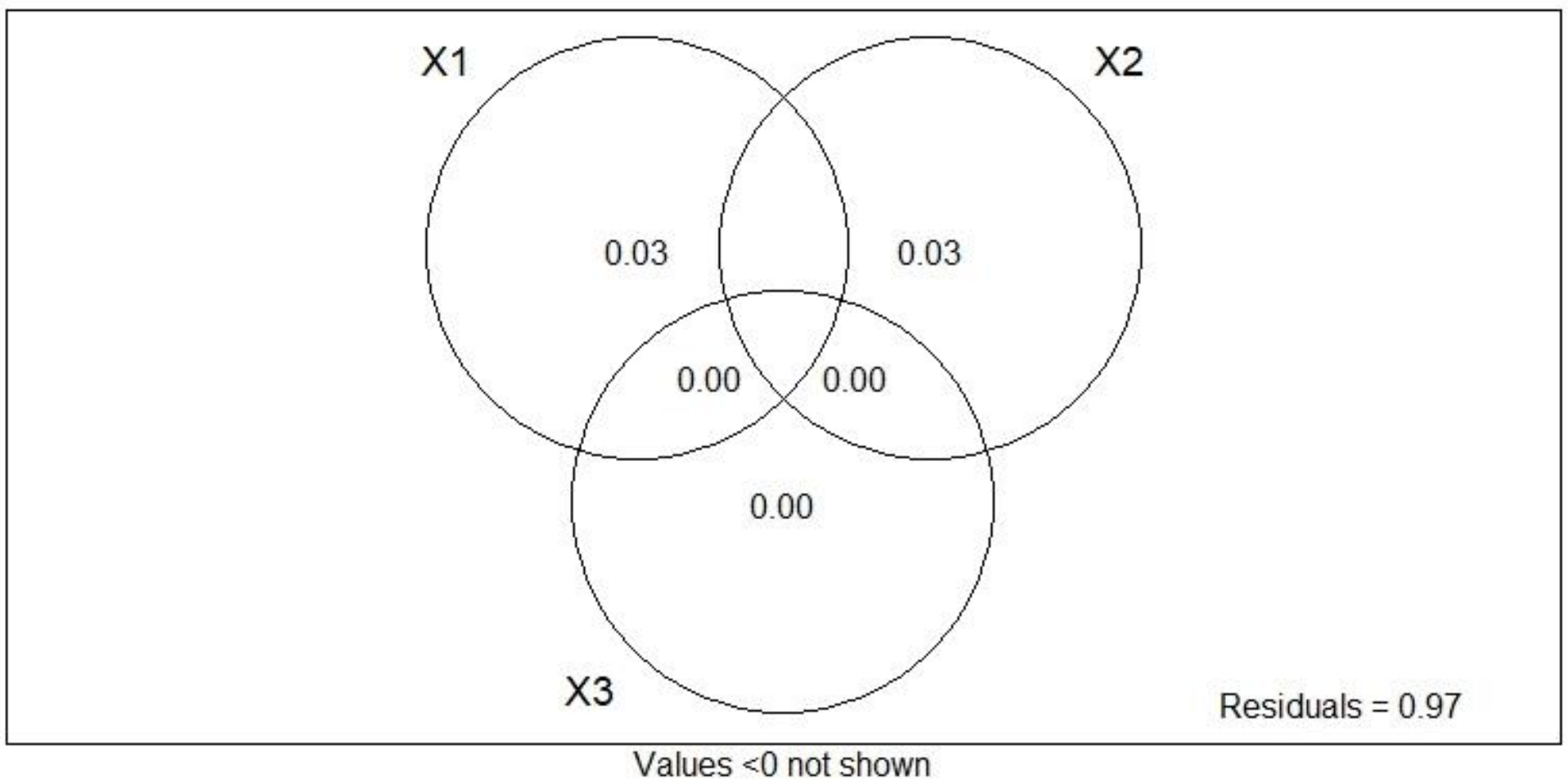

Figure S10 Variation partition to analyze the impact of content of carbon (C) and nitrogen (N) in the wood material and the resulting C/N ratio on bacterial community composition (X1 = C, X2 = N, X3 = C/N ratio)

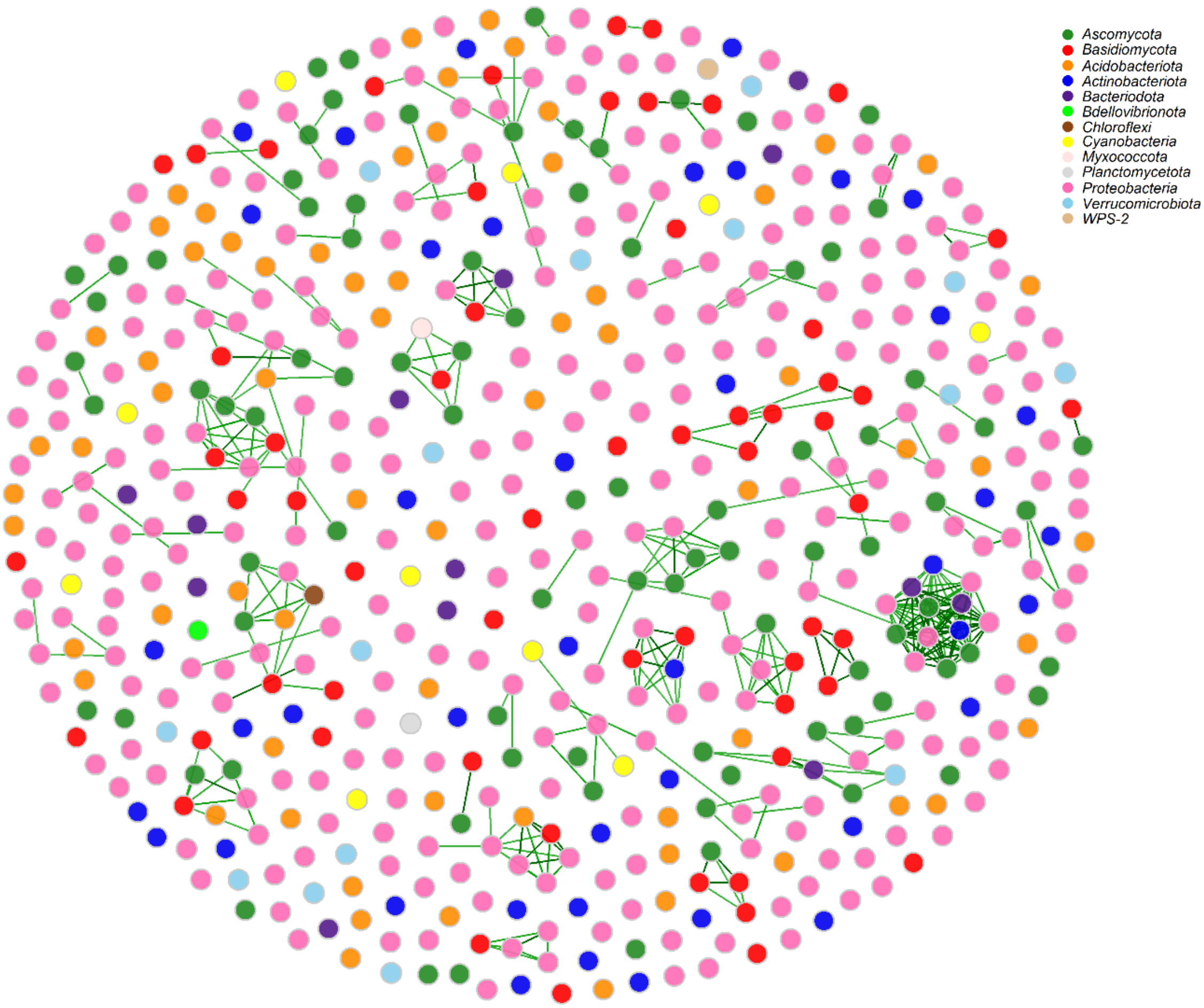

100 **Figure S11** Cross-domain network constructed of fungal and bacterial ASV with more than 1,500 counts. Pearson correlation coeffcient greater than 0.75 are indicated by green lines.
